# *Sgpl1* deletion elevates S1P levels, contributing to NPR2 inactivity and p21 expression that block germ cell development

**DOI:** 10.1038/s41419-021-03848-9

**Published:** 2021-06-03

**Authors:** Feifei Yuan, Zhijuan Wang, Yanli Sun, Hongwei Wei, Yanying Cui, Zhanying Wu, Chunyu Zhang, Ke-Ping Xie, Fengchao Wang, Meijia Zhang

**Affiliations:** 1 State Key Laboratory for Agrobiotechnology, College of Biological Sciences, China Agricultural University, 100193 Beijing, China; 2 Division of Cell, Developmental and Integrative Biology, School of Medicine, South China University of Technology, 510006 Guangzhou, China; 3 National Institute of Biological Sciences, 102206 Beijing, China

**Keywords:** Reproductive biology, Infertility

## Abstract

Sphingosine phosphate lyase 1 (SGPL1) is a highly conserved enzyme that irreversibly degrades sphingosine-1-phosphate (S1P). *Sgpl1*-knockout mice fail to develop germ cells, resulting in infertility. However, the molecular mechanism remains unclear. The results of the present study showed that SGPL1 was expressed mainly in granulosa cells, Leydig cells, spermatocytes, and round spermatids. *Sgpl1* deletion led to S1P accumulation in the gonads. In the ovary, S1P decreased natriuretic peptide receptor 2 (NPR2) activity in granulosa cells and inhibited early follicle growth. In the testis, S1P increased the levels of cyclin-dependent kinase inhibitor 1A (p21) and apoptosis in Leydig cells, thus resulting in spermatogenesis arrest. These results indicate that *Sgpl1* deletion increases intracellular S1P levels, resulting in the arrest of female and male germ cell development via different signaling pathways.

## Introduction

Normal gonad development is crucial for the production of mature gametes and the secretion of sexual hormones^1^. The follicle is the basic unit of the ovary. Follicular development initiates with the transition of the primordial stage to preantral follicles, and then these preantral follicles develop into large antral follicles under follicle-stimulating hormone (FSH) stimulation^2^. Natriuretic peptide type C (NPPC, also known as CNP) and its receptor natriuretic peptide receptor 2 (NPR2) maintain meiotic arrest of oocytes within full-grown follicles^3,4^. When luteinizing hormone (LH) surges occur, NPR2 guanylyl cyclase activity is decreased, and then oocytes resume meiosis^5,6^. On the other hand, NPPC/NPR2 is also essential for early follicle development. NPPC can promote early follicle growth by NPR2-produced cyclic guanosine monophosphate (cGMP)^7^, and *Npr2*-null mice exhibit small ovaries with very few antral follicles and infertility^8^. Morphogenesis of the testis begins shortly after birth and continues until puberty. Seminiferous tubules and Leydig cells are two key components of the testes. Seminiferous tubules are the site of spermatogenesis. Leydig cells secrete testosterone, which diffuses into the seminiferous tubules and promotes spermatogenesis. Mice with testosterone deficiency by *LH receptor* (*Lhcgr*) deletion or with *androgen receptor* (*Ar*) deletion exhibit spermatogenesis arrest^9,10^.

In recent years, many clinical cases have been reported that the mutation in sphingosine phosphate lyase 1 (SGPL1) gene can result in nephrotic syndrome and hypogonadism^11^. The most severe patients suffer from hydrops fetalis and/or perinatal death. Male infant patients exhibit a small penis, no palpable testes, low baseline testosterone levels, and/or high gonadotrophins^12^. Consistent with the human disease phenotype, SGPL1 deficiency in mice leads to the absence of spermatids by decreasing Leydig cell populations and testosterone production^13^. On the other hand, *Sgpl1* deletion also causes few antral follicles to develop in the ovary^13^. However, the molecular mechanisms of SGPL1 in germ cell development remain unclear. SGPL1 is an irreversible degradation enzyme that mediates cleavage of the lipid-signaling molecule sphingosine-1-phosphate (S1P)^14^. SGPL1 deficiency impairs its capacity to degrade S1P, resulting in high levels of S1P in the mouse liver^15^ and thymus^16^. In addition, the other two reversible S1P phosphatases, SGPP1 and SGPP2, can also regulate S1P levels^17^. S1P, a unique bioactive sphingolipid, is produced intracellularly by two sphingosine kinases: SphK1 and SphK2 (ref. ^18^). S1P plays a vital role in regulating cell proliferation, survival, movement, and calcium homeostasis. Extracellular S1P exerts its effects by binding to its specific G protein-coupled receptors (S1PRs) to regulate downstream signals^19,20^. S1P can also mobilize calcium release from the endoplasmic reticulum to mediate various cellular functions^21,22^. Our previous studies have indicated that S1P decreases NPR2 activity by elevating calcium levels in cumulus cells^5,23^. On the other hand, overexpression of SphK increases S1P levels and then enhances the transcription of *p21* in human MCF-7 breast cancer cells^24^. p21 can block cell proliferation in the G1 phase^25,26^, and promote cell apoptosis in granulocytes^27^.

This study focuses on the molecular mechanisms of SGPL1 in germ cell development. We found that deletion of *Sgpl1* elevated S1P levels. S1P accumulation decreased NPR2 activity to arrest follicle development in the ovaries and increased p21 expression and Leydig cell apoptosis to induce defects in spermatogenesis in the testes. Collectively, SGPL1 modulates S1P levels, which are crucial for germ cell development.

## Results

### The expression pattern of SGPL1 in mouse gonads

**Fig. 1:**
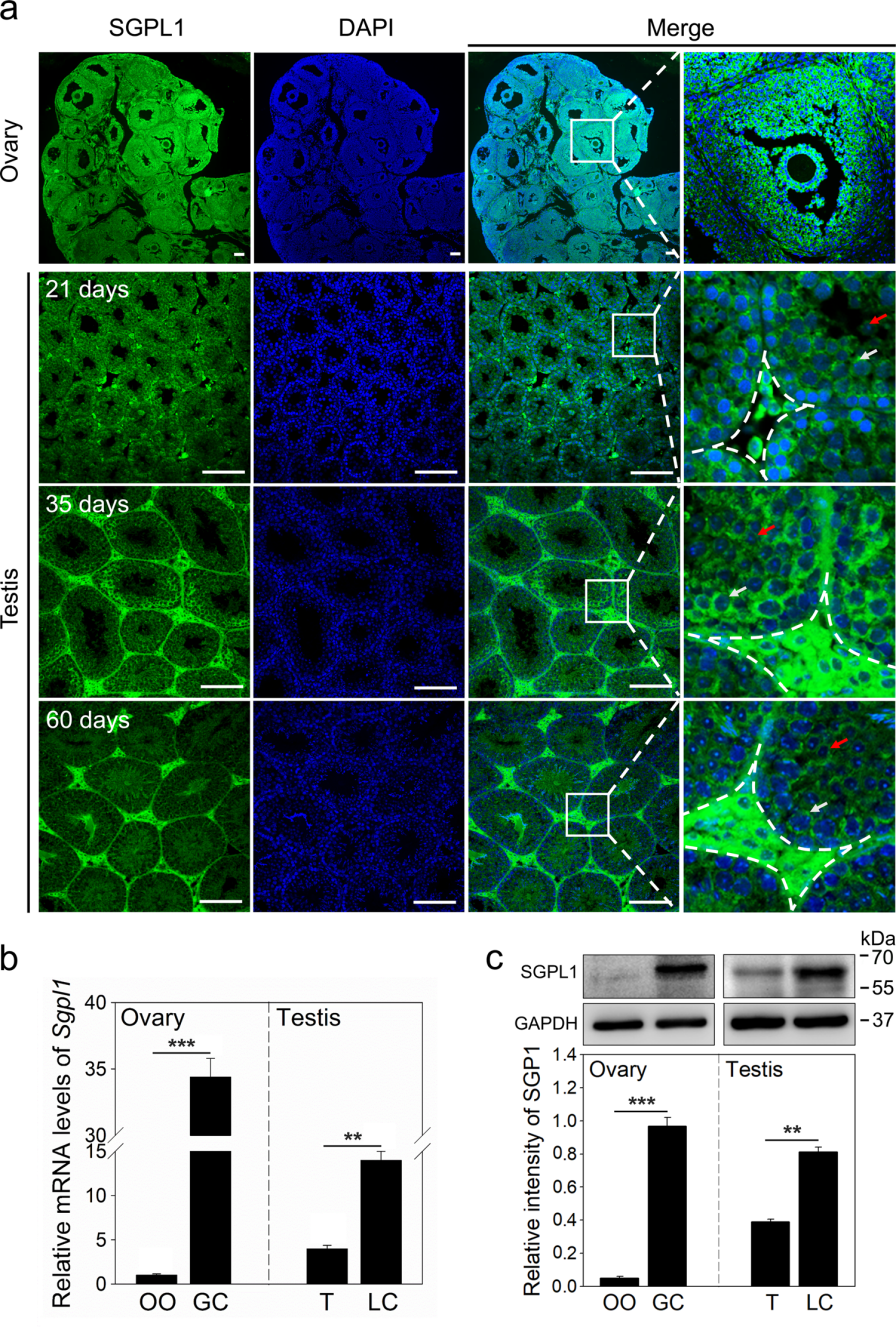
The expression pattern of SGPL1 in mouse gonads. **a** Immunofluorescence analysis of SGPL1 (green) in ovaries isolated from eCG-primed mice and in testes isolated from 21-, 35-, and 60-day-old mice. (*n* = 3 independent experiments). The nuclei were counterstained by DAPI (blue). The small white boxes indicate the locations of the enlarged areas, as shown in the following images. The cells in the dashed line box are Leydig cells. The white arrows show the spermatocytes, and the red arrows show the round spermatids. Scale bars: 100 μm. The mRNA (**b**) and protein (**c**) levels of SGPL1 in different cells and tissues isolated from 21-day-old mouse gonads (*n* = 4 independent experiments). GAPDH was used as a loading control. Bars indicate the mean ± SEM. **p < 0.01 and ***p < 0.001. OO oocyte, GC granulosa cell, T testis, LC Leydig cell.

We detected the expression pattern of SGPL1 in mouse gonads, since the mRNA levels of *Sgpl1* were significantly higher than those of *Sgpp1* and *Sgpp2* (Fig. S1). In the ovaries, SGPL1 was strongly localized in the cytoplasm of granulosa cells and theca cells but was slightly expressed in oocytes (Fig. 1a). In line with this, the mRNA and protein levels of SGPL1 were significantly higher in granulosa cells than in oocytes (Fig. 1b, c). In the testes, SGPL1 was strongly expressed in the cytoplasm of Leydig cells (verification with Leydig cell marker 3β-hydroxysteroid dehydrogenase, HSD3B), and was weakly expressed in peritubular myoid cells (Figs. 1a and S2). SGPL1 was also localized in the cytoplasm of germ cells, especially in spermatocytes and round spermatids (Fig. 1a). Consistent with this, the mRNA and protein levels of SGPL1 were significantly higher in Leydig cells than in whole testes (Fig. 1b, c). These results indicate that SGPL1 is mainly expressed in the cytoplasm of granulosa cells and Leydig cells.

### *Sgpl1* deletion increases S1P levels and disrupts gonad development

**Fig. 2:**
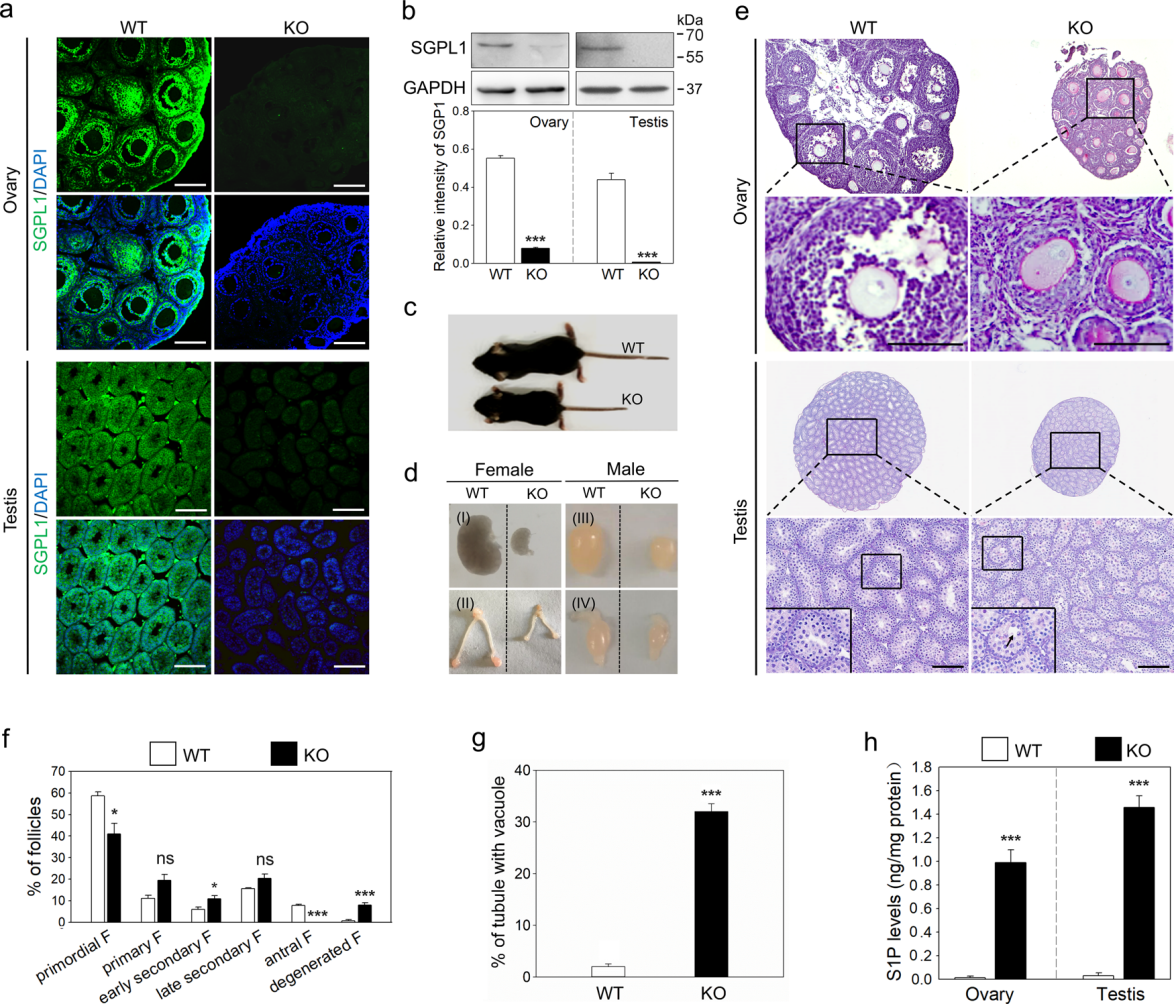
The effects of *Sgpl1* deletion on S1P levels and gonad development. Detection of SGPL1-knockout efficiency in 14-day-old ovaries and 21-day-old testes by immunofluorescence (**a**) and western blotting (**b**). (*n* = 3 independent experiments). The nuclei were counterstained by DAPI (blue). GAPDH was used as a loading control. Scale bars: 100 μm. Gross morphology of 21-day-old WT and KO mice (**c**) and gonads and deputy gonads (**d**) (I, ovary; II, uterus; III, testis; IV, epididymis; *n* = 3 independent experiments). **e** Histological analysis of 21-day-old ovaries and 25-day-old testes from WT and KO mice. (*n* = 3 independent experiments). The small black boxes indicate the enlarged areas, as shown in the following images. The black arrow shows the vacuole in the tubules. Scale bars: 100 μm. **f** Quantification of the numbers of follicles of different types in ovaries from WT and KO mice. Serial sections were used for the count. (*n* = 3 independent experiments. The total number of six ovaries was used in each group). F follicles. **g** The percentage of tubules with vacuoles in testes from WT and KO mice. (*n* = 3 independent experiments. The total number of 600 tubules was scored in each group). **h** The levels of S1P in ovaries and testes from WT and KO mice (*n* = 3 independent experiments. The total number of 24 ovaries or testes was used in each group). Bars indicate the mean ± SEM. ns no significance, *p < 0.05 and ***p < 0.001 vs. the WT group.

*Sgpl1* deletion leads to failure of follicular development and spermatogenesis^13^. To study the mechanisms of SGPL1 in germ cell development, we generated *Sgpl1*-knockout (KO) mice (Fig. S3). The KO efficiency was confirmed by immunofluorescence and western blotting (Fig. 2a, b). Consistent with a previous report^13^, *Sgpl1* KO mice exhibited a small body, uterus, ovary, and testis (Fig. 2c, d). In 21-day-old female KO mice, only preantral follicles, including primordial and secondary follicles, existed in ovaries, and 7.97 ± 1.12% of preantral follicles were atresic (Fig. 2e, f). However, well-developed follicles, including antral follicles, were present in the ovaries of wild-type (WT) mice (Fig. 2e, f). In 25-day-old male KO mice, the testis interstitium and gaps in the spermatocyte layers of the testis cords were decreased. The round spermatids were barely observed in the KO mice, indicating that germ cells were arrested at spermatocyte stage (Fig. 2e). Moreover, 32 ± 1.54% of tubules had vacuoles of varying sizes (Fig. 2e, g). In some tubules, spermatocytes exhibited sloughing (Fig. 2e). Thus, spermatogenesis does not occur normally. SGPL1 is the enzyme that irreversibly cleaves S1P^14^. Indeed, the levels of S1P in ovaries and testes from KO mice were dramatically increased compared with those from WT mice (Figs. 2h and S10). SGPL1 is expressed in granulosa cells, Leydig cells, male germ cells, and peritubular myoid cells, suggesting that *Sgpl1* deletion elevates S1P levels possibly due to the accumulation of S1P in these cells.

### *Sgpl1* deletion decreases NPR2 activity and increases p21 expression in the ovary

**Fig. 3:**
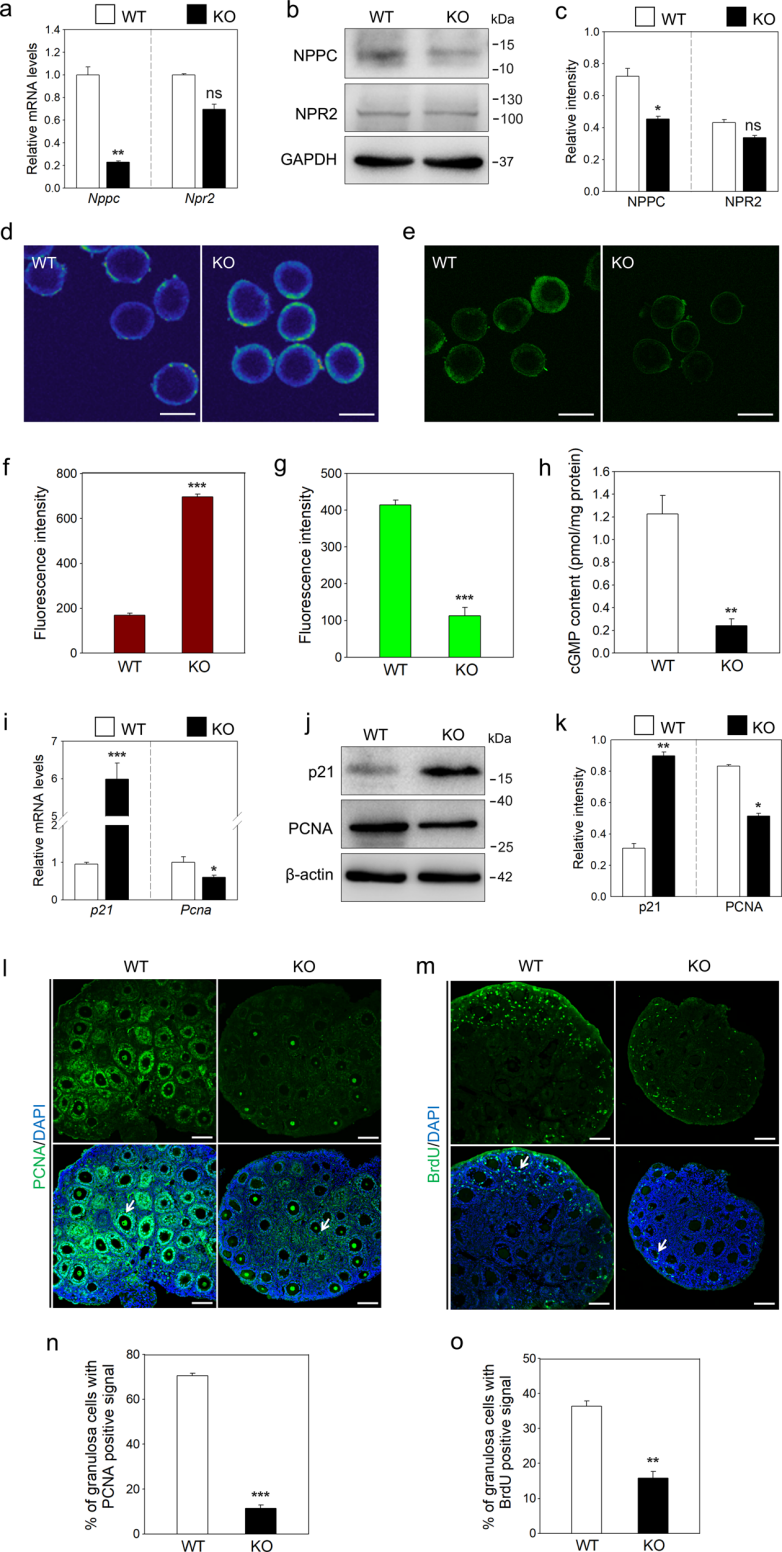
The effects of *Sgpl1* deletion on NPR2 activity and p21 expression in the ovary. The mRNA (**a**) and protein (**b**, **c**) levels of NPPC and NPR2 in ovaries of WT and KO mice. (*n* = 3 independent experiments). GAPDH was used as a loading control. **d** Fluorescence images of calcium in granulosa cells after incubation with Fluo 3-AM (pseudocolor). **f** Statistical analysis of the calcium levels. The fluorescence intensity indicates the mean ± SEM. (*n* = 3 independent experiments. The total number of 21 follicles was scored in each group). **e** Fluorescence images of the binding affinity of NPR2 for NPPC in granulosa cells after incubation with FAM-NPPC (green). **g** Statistical analysis of the binding affinity. The fluorescence intensity indicates the mean ± SEM. (*n* = 3 independent experiments. The total number of 21 follicles was scored in each group). **h** Cyclic GMP levels in ovaries of WT and KO mice (*n* = 3 independent experiments. The total number of 12 ovaries was used in each group). The mRNA (**i**) and protein levels (**j**, **k**) of p21 and PCNA in ovaries of WT and KO mice (*n* = 3 independent experiments). β-actin was used as a loading control. Immunofluorescence analysis of PCNA (**l**) and BrdU (**m**) in ovaries of WT and KO mice (green). (*n* = 3 independent experiments). Arrows show the positive granulosa cells. The nuclei were counterstained by DAPI (blue). Quantitative analysis of granulosa cells positive for PCNA (**n**) and BrdU (**o**) in WT and KO mice. The percentage of positive granulosa cells in secondary follicles was counted. (*n* = 3 independent experiments. The total number of 36 secondary follicles was scored in each group). Scale bars: 100 μm. Bars indicate the mean ± SEM. ns no significance, *p < 0.05, **p < 0.01 and ***p < 0.001 vs. the WT group.

*Sgpl1* deletion leads to the accumulation of S1P in the ovary. S1P can inactivate NPR2 in cumulus cells by elevating calcium levels^5,23^, and *Npr2* KO mice exhibit the arrest of early follicle development^8,13^. In the present study, the calcium fluorescence intensity (pseudocolor) was significantly increased and the binding affinity of NPR2 for NPPC (green color) was significantly decreased in granulosa cells of KO mice (Fig. 3d–g). In addition, *Sgpl1* deletion also significantly decreased the mRNA and protein levels of NPPC, which further decreased NPR2 function (Fig. 3a–c). Consistent with these findings, the cGMP levels in ovaries from KO mice were significantly decreased compared with those from WT mice (Fig. 3h). These findings indicate that deletion of *Sgpl1* decreases NPR2 activity, which could lead to the arrest of early follicle development.

We further studied the effects of *Sgpl1* deletion on cell proliferation and apoptosis in the ovary. The proliferating cell nuclear antigen (PCNA)-, BrdU-, and Ki-67-positive granulosa cells in KO mice were significantly decreased compared with those in WT mice (11.43 ± 1.42% vs. 70.43 ± 1.06%, 15.76 ± 1.9% vs. 36.36 ± 1.5% and 8.90 ± 1.14% vs. 49.06 ± 3.83%, respectively; Figs. 3l–o and S4a, c). Consistent with this, the mRNA and protein levels of PCNA in the ovaries of KO mice were significantly lower than those in the ovaries of WT mice (Fig. 3i–k). TUNEL-positive signals were slightly increased in the theca cells of KO mice (Fig. S4b). However, *Casepase-3* mRNA level and cleaved caspase-3 protein level in the ovaries were not different between WT and KO mice (Fig. S4d, e). p21, a factor that inhibits cell proliferation^25,26^, was mainly expressed in granulosa cells (Fig. S5). Deletion of *Sgpl1* significantly increased the mRNA and protein levels of p21 in the ovaries (Fig. 3i–k). These findings indicate that deletion of *Sgpl1* inhibits the proliferation of granulosa cells, and the increase in p21 expression may be involved in this process.

### S1P decreases NPR2 activity and inhibits preantral follicle development in vitro

**Fig. 4:**
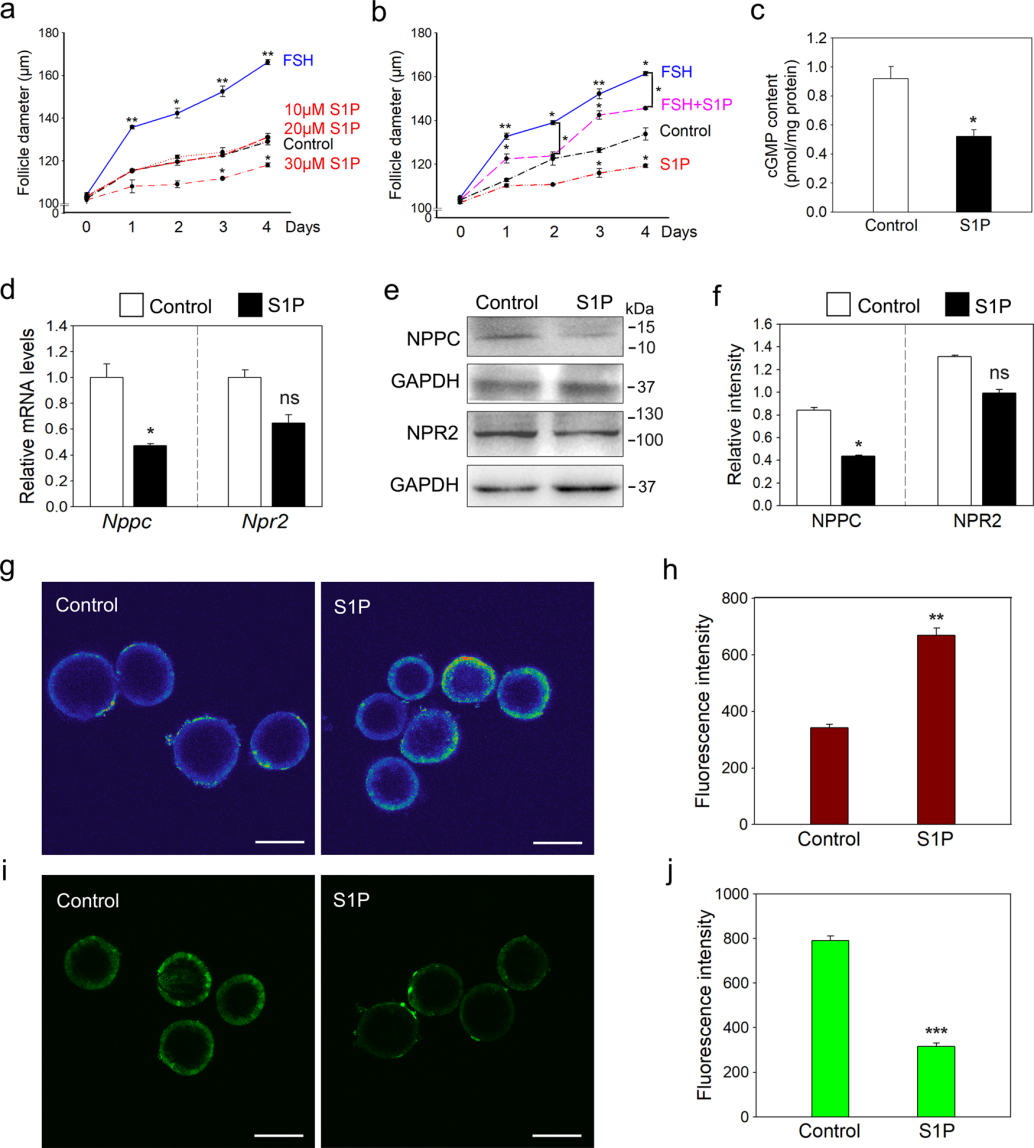
The effects of S1P on NPR2 activity and preantral follicle development. **a** The effect of S1P (10, 20, 30 μM) or FSH (25 ng/ml) on preantral follicle growth. *p < 0.05 and **p < 0.01 compared with the corresponding control. (*n* = 3 independent experiments. The total number of 36 secondary follicles was used in each group). **b** The effect of S1P on FSH-induced follicle growth. Preantral follicles were treated with FSH (25 ng/ml) and/or S1P (30 μM) for 4 days, and the diameters of follicles were measured daily. *p < 0.05 and **p < 0.01 compared with the corresponding control. The diameters in the S1P + FSH group were significantly decreased compared with those in the corresponding FSH group. *p < 0.05. (*n* = 3 independent experiments. The total number of 36 secondary follicles was used in each group). **c** Cyclic GMP levels in ovaries from the control and S1P treatment groups (*n* = 3 independent experiments. The total number of 12 ovaries was used in each group). The mRNA (**d**) and protein (**e**, **f**) levels of NPPC and NPR2 in ovaries from the control and S1P treatment groups (*n* = 3 independent experiments). GAPDH was used as a loading control. Fluorescence images (**g**) and statistical analysis (**h**) of calcium in granulosa cells after incubation with Fluo 3-AM (pseudocolor). The fluorescence intensity indicates the mean ± SEM. (*n* = 3 independent experiments. The total number of 30 follicles was scored in each group). Fluorescence images (**i**) and statistical analysis (**j**) of the binding affinity of NPR2 for NPPC in granulosa cells after incubation with FAM-NPPC (green). The fluorescence intensity indicates the mean ± SEM. (*n* = 3 independent experiments. The total number of 21 follicles was scored in each group). Scale bars: 100 μm. Bars indicate the mean ± SEM. ns no significance, *p < 0.05, **p < 0.01 and ***p < 0.001 vs. the control group.

We studied the effect of S1P on early follicle development. Compared with the control group, treatment with S1P (30 μM) led to a significant decrease in follicle size (Fig. 4a). Furthermore, the FSH-induced growth of preantral follicles could be reversed by the addition of S1P (Figs. 4b and S6). Next, we studied the effect of S1P on NPR2 activity. Compared with the control group, treatment with S1P for 2 h significantly increased the calcium levels and decreased the binding affinity of NPR2 for NPPC in granulosa cells of preantral follicles (Fig. 4g–j). Moreover, S1P also reduced the mRNA and protein levels of NPPC in cultured ovaries (Fig. 4d–f). In line with these findings, the cGMP levels in the ovaries of S1P treatment were significantly decreased compared with that in control (Fig. 4c). These findings indicate that the addition of S1P can decrease the function of NPR2, and result in the arrest of early follicle growth.

### *Sgpl1* deletion increases p21 expression and cell apoptosis in the testis

**Fig. 5:**
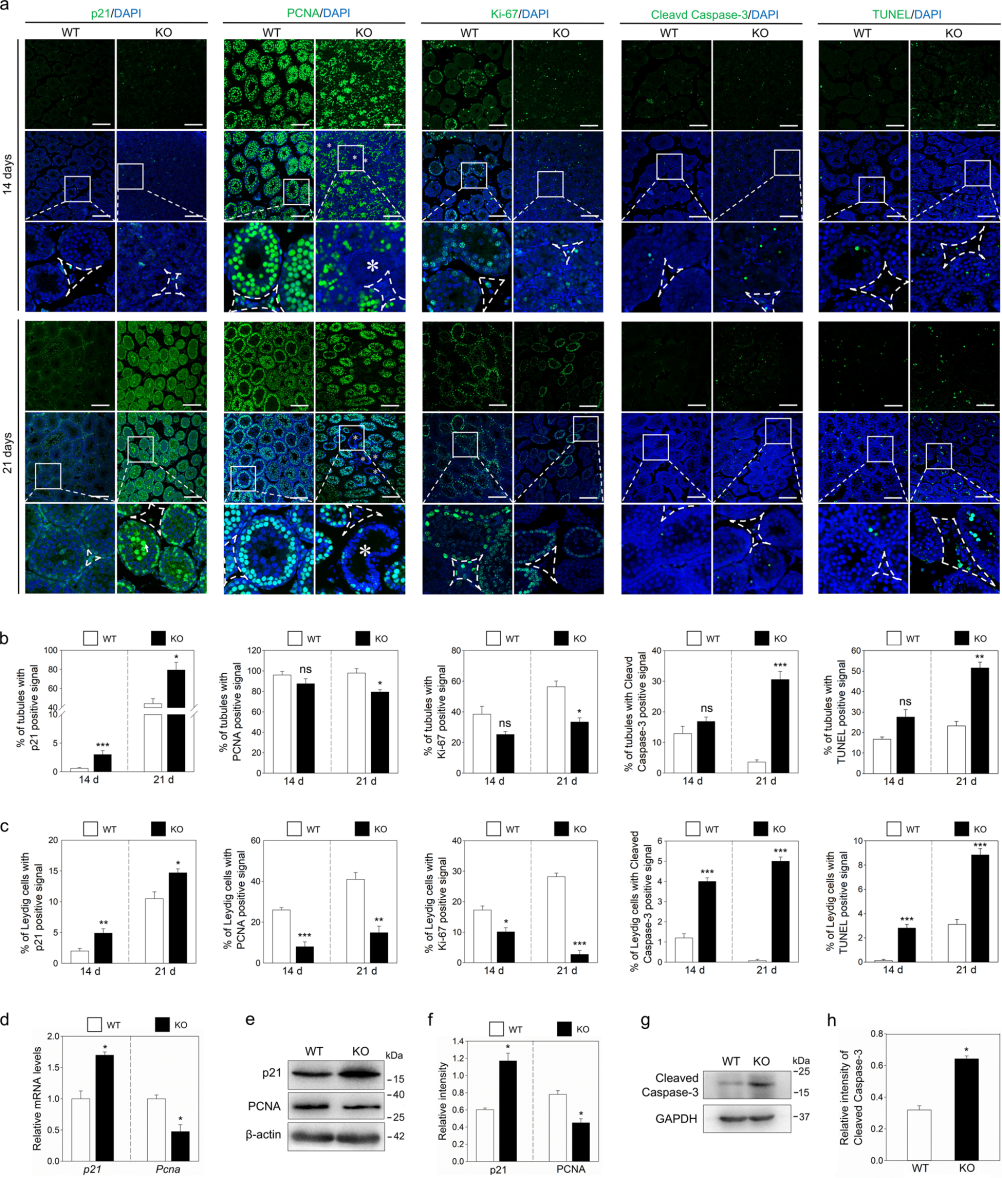
The effects of *Sgpl1* deletion on p21 expression and apoptosis in the testis. **a** Immunofluorescence analysis of p21, PCNA, Ki-67, cleaved caspase-3, and TUNEL (green) in WT and KO testes from 14- and 21-day-old mice. (*n* = 3 independent experiments). The nuclei were counterstained by DAPI (blue). The small white boxes indicate the location of the enlarged areas, as shown in the following images. The cells in the dashed line box are Leydig cells. Scale bars: 100 μm. Asterisks (*) indicate the seminiferous tubules with few PCNA-positive signals. **b** The percentage of seminiferous tubules with p21-, PCNA-, Ki-67-, cleaved caspase-3- and TUNEL-positive signals in WT and KO testes from 14- and 21-day-old mice. (*n* = 3 independent experiments. The total number of 450 tubules was scored in each group). **c** The percentage of Leydig cells with p21-, PCNA-, Ki-67-, cleaved caspase-3- and TUNEL-positive signals in WT and KO testes from 14- and 21-day-old mice. (*n* = 3 independent experiments. The total number of 1000 Leydig cells was scored in each group). The mRNA (**d**) and protein (**e**, **f**) levels of p21 and PCNA in testes from 21-day-old WT and KO mice. (*n* = 3 independent experiments). β-actin was used as a loading control. **g**, **h** The protein levels of cleaved caspase-3 in testes from 21-day-old WT and KO mice. (*n* = 3 independent experiments). GAPDH was used as a loading control. Bars indicate the mean ± SEM. ns no significance, *p < 0.05, **p < 0.01 and ***p < 0.001 vs. the WT group.

*Npr2* deletion had no effect on testis development^8^. Therefore, we studied the effect of *Sgpl1* deletion on p21 expression. p21 was expressed in the nuclei of Leydig cells and spermatocytes (Fig. 5a). The positive signals of p21 in testes from KO mice were significantly increased compared with those from WT mice (Fig. 5b, c). Also, the mRNA and protein levels of p21 in testes from KO mice were significantly higher than those from WT mice (Fig. 5d–f). We further studied the effect of *Sgpl1* deletion on cell proliferation in testes from 14- and 21-day-old mice. The results showed that the percentage of tubules with PCNA- and Ki-67-positive signals in KO testes was decreased at 14 days compared with that in WT testes (87.5% vs. 96% and 25.3% vs. 38.4%, respectively; Fig. 5a, b), and was further decreased at 21 days (79.4% vs. 98% and 33.3% vs. 56.4%, respectively; Fig. 5a, b). The percentage of Leydig cells with PCNA- and Ki-67-positive signals in KO testes was significantly decreased at 14 days compared with that in WT testes (8% vs. 26% and 10.1% vs. 17.2%, respectively; Fig. 5a, c), and was further decreased at 21 days (14.9% vs. 41% and 2.7% vs. 28.2%, respectively; Fig. 5a, c). Consistent with this, the mRNA and protein levels of PCNA in the testes of KO mice were significantly lower than those of WT mice (Fig. 5d–f).

We also studied the effect of *Sgpl1* deletion on cell apoptosis in the testes. The percentage of tubules with cleaved caspase-3- and TUNEL-positive signals was increased in testes from 14-day-old KO mice compared with those from 14-day-old WT mice (16.87% vs. 12.9% and 27.58% vs. 16.7%, respectively; Fig. 5a, b), and the increase was more obvious at 21 days (30.5% vs. 3.59% and 51.5% vs. 23.24%, respectively; Fig. 5a, b). The percentage of Leydig cells with cleaved caspase-3- and TUNEL-positive signals in KO testes was significantly increased at 14 days compared with that in WT testes (4% vs. 1.2% and 2.84% vs. 0.13%, respectively; Fig. 5a, c), and the increase was more obvious at 21 days (5% vs. 0.08% and 8.83% vs. 3.1%, respectively; Fig. 5a, c). In line with this, *Sgpl1* deletion significantly increased the cleaved caspase-3 protein level in the testes (Fig. 5g, h). These results suggest that *Sgpl1* deletion leads to an increase in p21 expression and cell apoptosis.

### S1P increases p21 expression and cell apoptosis in Leydig cells

**Fig. 6:**
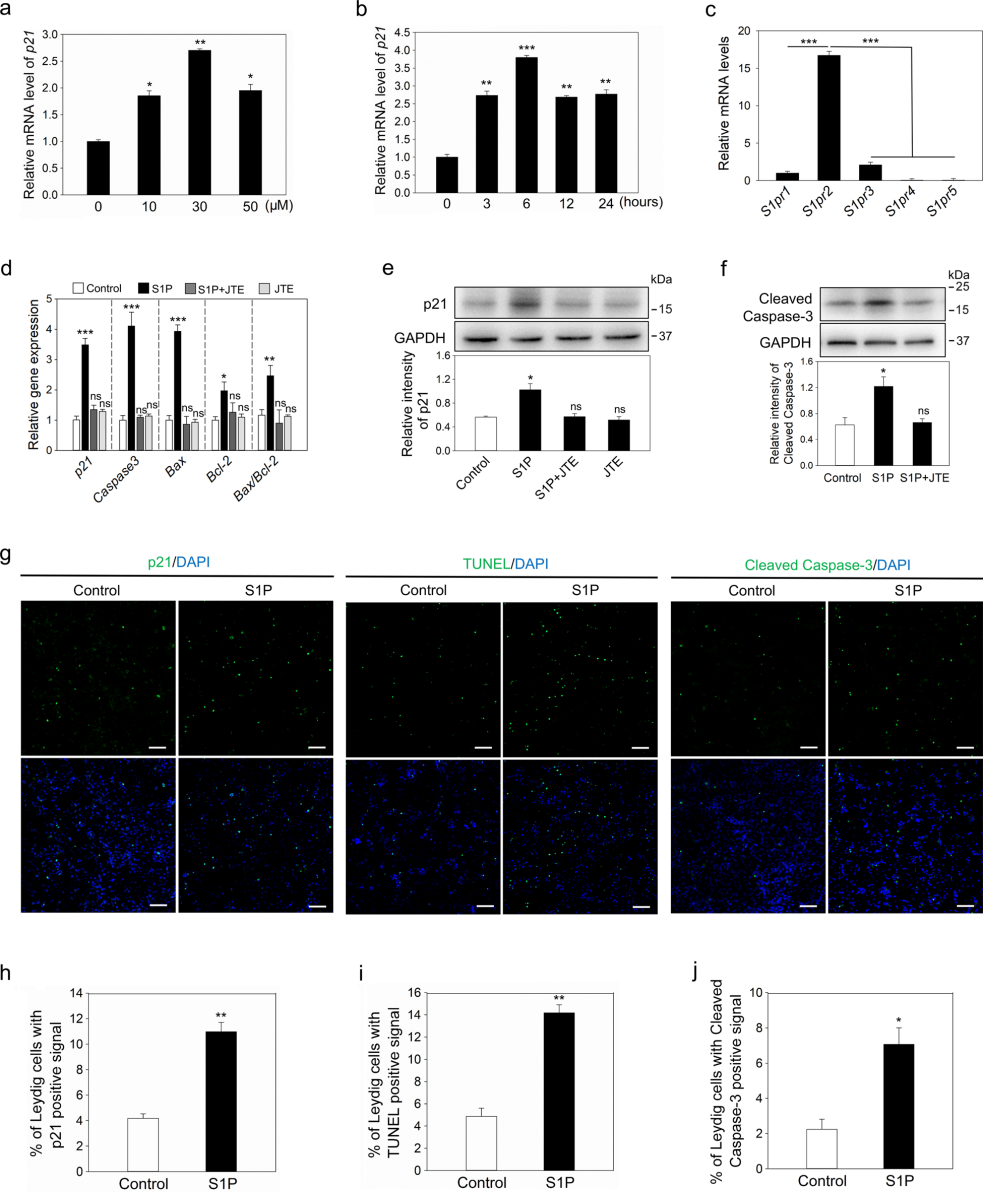
The effect of S1P on p21 expression and apoptosis in Leydig cells. **a** The mRNA levels of *p21* were measured after incubation of Leydig cells with different concentrations of S1P (0, 10, 30, 50 μM) for 24 h. (*n* = 3 independent experiments). **b** The mRNA levels of *p21* were measured after incubation of Leydig cells with 30 μM S1P for 0, 3, 6, 12 or 24 h. (*n* = 3 independent experiments). **c** The mRNA levels of *S1prs* in Leydig cells. (*n* = 3 independent experiments). **d** The mRNA levels of *p21*, *caspase-3*, *Bax,* and *Bcl-2* were measured after incubation of Leydig cells with S1P (30 μM) and/or JTE-013 (7.5 μM) for 6 h. (*n* = 3 independent experiments). The protein levels of p21 (**e**) and cleaved caspase-3 (**f**) were measured after incubation of Leydig cells with S1P (30 μM) and/or JTE-013 (7.5 μM) for 6 h. (*n* = 3 independent experiments). GAPDH was used as a loading control. **g** Immunofluorescence analysis of p21, TUNEL, and cleaved caspase-3 (green) in Leydig cells after incubation without or with S1P (30 μM) for 6 h. (*n* = 3 independent experiments). The nuclei were counterstained by DAPI (blue). Scale bars: 100 μm. The percentage of Leydig cells with p21 (**h**), TUNEL (**i**), and cleaved caspase-3 (**j**) positive signals. (*n* = 3 independent experiments. The total number of 6000 Leydig cells was scored in each group). Bars indicate the mean ± SEM. ns no significance, *p < 0.05, **p < 0.01 and ***p < 0.001 vs. the control group. JTE: JTE-013, an inhibitor of S1PR2.

We studied the effect of S1P on p21 expression in immature Leydig cells. S1P significantly increased the *p21* mRNA level in a dose-dependent manner, reaching a maximum at 30 μM (Fig. 6a). The temporal dynamics curve showed that S1P could significantly increase the mRNA levels of *p21* as early as 3 h, reaching a maximum at 6 h (Fig. 6b). In line with this, S1P significantly increased p21 protein level (Fig. 6e) and p21-positive Leydig cells compared with the control (10.96 ± 0.73% vs. 4.18 ± 0.35%, Fig. 6g, h). The effect of S1P on cell apoptosis was also studied. S1P significantly increased the mRNA levels of *Caspase-3*, *Bax*, and *Bcl-2* (Fig. 6d). Consistent with this, the protein levels of cleaved caspase-3 were significantly increased after S1P treatment (Fig. 6f). Furthermore, the percentage of Leydig cells with TUNEL- and cleaved caspase-3-positive signals in the S1P treatment group was significantly increased compared with that in the control group (14.18 ± 0.71% vs. 4.86 ± 0.74% and 7.07 ± 0.93% vs. 2.24 ± 0.57%, respectively; Fig. 6g, i, j).

The role of S1P receptors was studied. The mRNA levels of *S1pr2* were significantly higher than that of *S1pr1* and *S1pr3*, and the mRNA levels of *S1pr4* and *S1pr5* were almost not detected (Fig. 6c). Furthermore, S1P-promoted p21 expression was inhibited by the S1PR2 inhibitor JTE-013 in a dose-dependent manner and was completely blocked by JTE-013 at 7.5 μM (Figs. 6d, e and S8a), while S1PR1/3 inhibitor VPC23019 had a slightly inhibitory effect on S1P-promoted p21 expression (Fig. S8b). JTE-013 also completely blocked S1P-promoted expression of *Caspase-3*, *Bax*, *Bcl-2* mRNA (Fig. 6d) and cleaved caspase-3 protein (Fig. 6f). These results indicate that S1P increases p21 expression and apoptosis in Leydig cells through S1PR2.

### *Sgpl1* deletion changes the transcriptomic integrity in mouse gonads

**Fig. 7:**
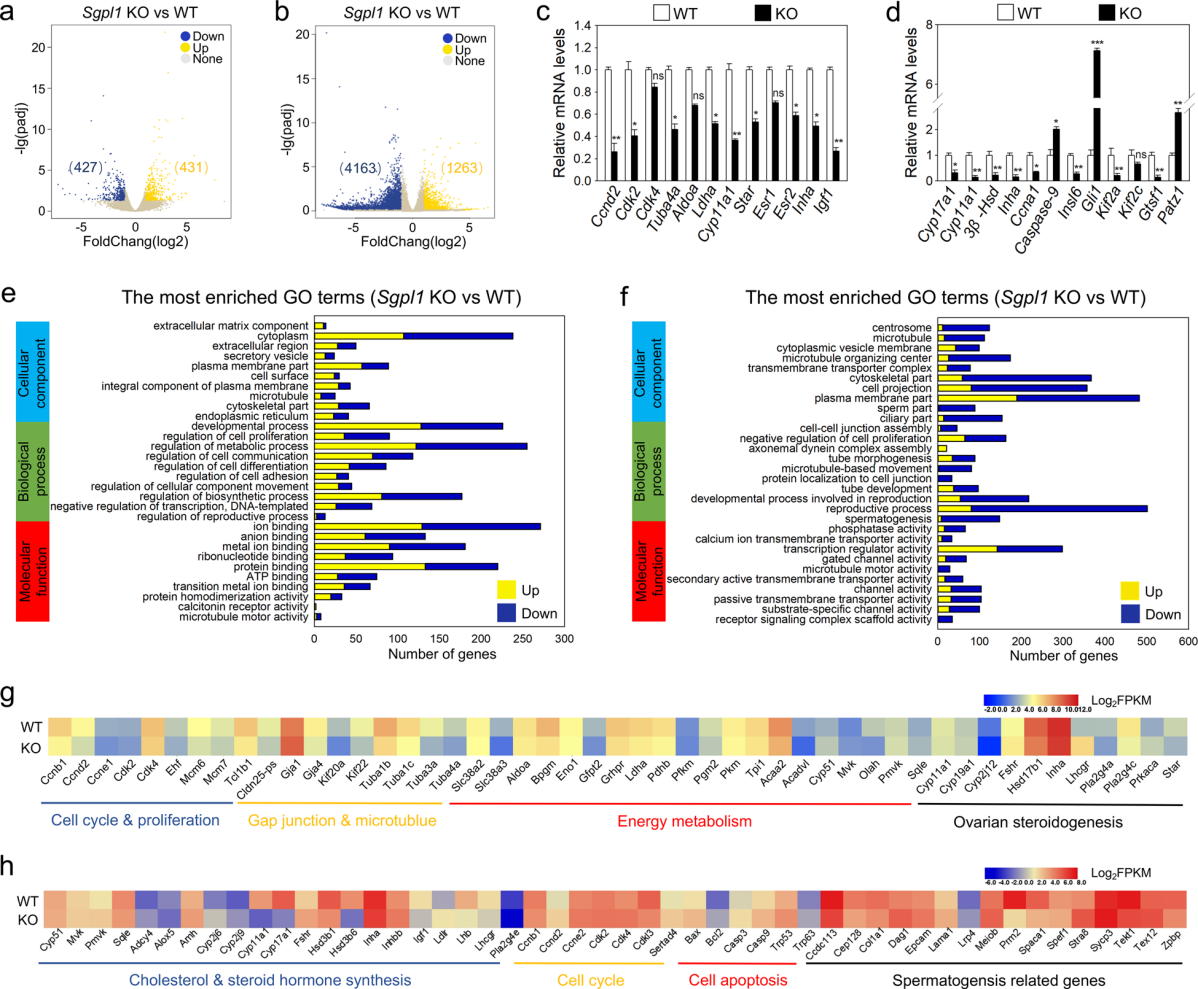
The effect of *Sgpl1* deletion on the integrity of transcriptome. Volcano plot illustrating the differentially expressed transcripts in granulosa cells (**a**) and testes (**b**). Down downregulated in KO mice compared with WT mice, Up upregulated in KO mice compared with WT mice, None no differences in KO mice compared with WT mice. **c**, **d** The changes in the expression of representative transcripts selected from the RNA-seq data were validated by qRT-PCR. (*n* = 4 independent experiments). Bars indicate the mean ± SEM. ns no significance, *p < 0.05, **p < 0.01 and ***p < 0.001 vs. the WT group. GO analysis of the differentially expressed genes in granulosa cells (**e**) and testes (**f**). **g** Heat map illustrating differences between WT and KO granulosa cells in the expression of a group of transcripts involved in various processes. **h** Heat map illustrating differences between WT and KO testes in the expression of a group of transcripts involved in various processes.

We applied RNA sequencing (RNA-seq) technology to further investigate the molecular mechanisms of SGPL1 in mouse germ cell development. In the ovary, a total of 858 transcripts, including 431 upregulated and 427 downregulated transcripts, were differentially expressed in granulosa cells of KO mice compared with those of WT mice (Fig. 7a and Table S3). The changes in the expression of representative transcripts were validated by qRT-PCR (Fig. 7c) and/or western blotting (Fig. S9a, b). Gene Ontology (GO) terms associated with differentially expressed transcripts were mainly enriched in three aspects: cellular component, biological process, and molecular function (Fig. 7e). Further analysis of gene-enrichment found that some changed transcripts of *Sgpl1* KO mice could impair granulosa cell development. These include genes controlling the cell cycle, ovarian steroidogenesis, gap junctions, and energy metabolism (Fig. 7g). For example, downregulation of cell cycle genes (i.e., *Ccnd2* and *Cdk2*) and cell growth genes (i.e., *Igf1*) could potentially arrest granulosa cell proliferation. In addition, downregulation of tubulin genes (i.e., *Tuba1b* and *Tuba4a*) could potentially impair cellular structure and gap junction formation in granulosa cells^28^. On the other hand, downregulation of genes associated with glycolysis/gluconeogenesis (i.e., *Ldha*), amino acid transport (i.e., *Slc38a3*), and fatty acid metabolism (i.e., *Olah*) could potentially impair granulosa cell nutrition and energy metabolism. Thus, deletion of *Sgpl1* could potentially impair the growth and metabolism of granulosa cells, which could participate in the arrest of follicle development.

In the testis, a total of 5426 transcripts, including 1263 upregulated and 4163 downregulated transcripts, were differentially expressed in KO mice compared with WT mice (Fig. 7b and Table S4). Then, the changes in the expression of representative transcripts were validated by qRT-PCR (Fig. 7d) and/or western blotting (Fig. S9c, d). GO terms were also mainly enriched in cellular component, biological process, and molecular function (Fig. 7f). Further analysis of gene-enrichment showed that some of the transcriptomic changes in *Sgpl1* KO testes could impair testis development (Fig. 7h). These include genes controlling the cell cycle, cell apoptosis, steroidogenesis, and spermatogenesis. For example, downregulation of cell cycle-related genes (i.e., *Ccnb1* and *Ccnd2*) and upregulation of cell apoptosis-related genes (i.e., *Bax*, *Bcl-2*, and *Caspase-3*) could potentially increase the apoptosis of Leydig cells and spermatocytes. In addition, downregulation of genes associated with steroid hormone synthesis (i.e., *Lhcgr*, *Cyp17a1*, *Cyp11a1,* and *Hsd3b6*) could potentially impair testosterone production. On the other hand, downregulation of spermatogenesis-associated genes (i.e., *Kif2a*, *Gtsf1*, and *Insl6*) and upregulation of genes associated with spermatogenesis suppression (i.e., *Patz1* and *Gli1*) could potentially impair spermatogenesis. Thus, these changed transcripts in *Sgpl1* KO testes could participate in the arrest of spermatogenesis.

## Discussion

SGPL1 is the key regulatory enzyme in the sphingolipid degradative pathway^14^. *Sgpl1* KO mice are unable to produce mature germ cells and exhibit infertility^13^. In the present study, *Sgpl1* deletion led to the accumulation of S1P in the gonads. In the ovary, the increase in S1P decreased NPR2 activity in granulosa cells and inhibited early follicle development. In the testis, the increase in S1P promoted p21 expression and cell apoptosis in Leydig cells. The decrease in Leydig cell populations and testosterone levels leads to failure in spermatogenesis^13^. Therefore, SGPL1 plays a vital role in the development of germ cells by regulating S1P levels.

Disruption of the dynamic balance in cellular S1P leads to defects in reproduction^29^. In our study, SGPL1 was the main S1P degradation enzyme in the gonads. Furthermore, deletion of *Sgpl1* led to S1P accumulation in the ovaries and testes, consistent with a previous report that the inhibition of SGPL1 extremely increases S1P levels in the mouse liver^15^. Thus, SGPL1 plays an important role in regulating intracellular S1P levels in the gonads. S1P acts as a phospholipid signaling molecule that can mobilize intracellular calcium release from the endoplasmic reticulum^23^ and then decrease NPR2 activity by reducing its binding affinity for NPPC in cumulus cells and vascular smooth muscle cells^5,30^. Interestingly, deletion of *Sgpl1* also led to calcium increase and NPR2 inactivity in granulosa cells. In vitro, S1P decreased the activity of NPR2 in granulosa cells by increasing intracellular calcium. On the other hand, S1P could directly decrease NPPC expression in the ovary to further decrease NPR2 activity. These results indicate that *Sgpl1* deletion decreases NPR2 activity in the ovary by accumulating S1P. It has been reported that knocking out NPR2 causes the arrest of early follicle development^8^. Thus, deletion of *Sgpl1* increases S1P levels, contributing to NPR2 inactivity that leads to follicle development failure.

Normal follicle development depends on the proliferation of granulosa cells. In the present study, we found that *Sgpl1* deletion led to a reduction in PCNA-, BrdU-, and Ki-67-positive granulosa cells, suggesting the inhibition of granulosa cell proliferation. NPPC/NPR2 acts through the cGMP signaling pathway and can promote the growth of cultured preantral follicles in mice and rats^7,31^. Therefore, the inactivity of NPR2 by *Sgpl1* deletion may reduce the proliferation of granulosa cells, resulting in the obstacle of early follicle growth. On the other hand, *Sgpl1* deletion increased the mRNA and protein levels of p21 in granulosa cells possibly by S1P, since S1P can increase the transcription of *p21* in human MCF-7 breast cancer cells^24^. The increase in p21 may participate in the arrest of granulosa cell proliferation. Furthermore, our RNA-seq data showed that deletion of *Sgpl1* downregulated the expression of tubulin in granulosa cells, which may impair gap junction formation by disturbing the function of connexin-43 (ref. ^28^). The downregulation of transcripts related to cell metabolic enzymes (Fig. 7g) might disrupt granulosa cell metabolism progression. These changed transcripts may also be involved in the arrest of early follicle development.

*Sgpl1* deletion decreases Leydig cell populations and testosterone levels, leading to failure in spermatogenesis^13^. In the present study, *Sgpl1* deletion resulted in an increase in S1P levels and Leydig cell apoptosis in the testes. In vitro, S1P significantly increased the apoptosis of Leydig cells by S1PR2. These results indicate that *Sgpl1* deletion increases the apoptosis of Leydig cells by S1P, resulting in the decrease of testosterone levels and then the arrest of spermatogenesis^13^. S1P may not affect spermatogenesis by NPR2 inactivity^8^. *Sgpl1* deletion increased p21 expression in the testes. In vitro, S1P increased p21 expression in Leydig cells by S1PR2, which is different from a previous report that S1P upregulates p21 expression independently of its cell surface receptors in human MCF-7 breast cancer cells^24^. These results indicate that *Sgpl1* deletion promotes p21 expression in the testes by S1P accumulation. It has been reported that p21 could promote granulocyte apoptosis^27^. Therefore, *Sgpl1* deletion increases Leydig cell apoptosis possibly by S1P-induced p21 expression. The reduction in Leydig cell populations results in a decrease in testosterone levels^32^. On the other hand, *Sgpl1* deletion also decreased the expression of *Cyp11a1*, *Lhcgr*, *Star*, and *Hsd3b6*, which further aggravated the reduction in testosterone production.

The reduction in Leydig cell populations and testosterone levels could result in germ cell apoptosis^10,33^. On the other hand, SGPL1 was also expressed in male germ cells, and *Sgpl1* deletion may increase S1P levels and p21 expression in these cells. This will aggravate the apoptosis of germ cells. Our RNA-seq data showed that the mRNA levels of *Kif2a*, *Gtsf1*, and *Insl6* (ref. ^34^) were decreased, and that the mRNA levels of *Patz1* and *Gli1* (ref. ^35^) were increased in *Sgpl1* KO mice, indicating spermatogenesis disorder. We used testicular tissue to measure the S1P levels because of the limited number of Leydig cells in *Sgpl1* KO mice. Anyway, the increase in S1P levels in the testicular microenvironment could act on Leydig cells. It is needed to further study S1P levels and SGPL1 functions in Leydig cells and germ cells, respectively.

In our study, *Sgpl1* deletion increased S1P levels and then inhibited early follicular development and spermatogenesis mainly by NPR2 inactivity and p21 expression, respectively. Thus, SGPL1 plays a crucial role in mouse germ cell development by regulating S1P levels. *Sgpl1* recessive mutations in humans cause primary gonadal failure^12^. Therefore, the mechanism of SGPL1 in mice provides a potential implication for the diagnosis and treatment of clinical infertility.

## Materials and methods

### Animals and chemicals

ICR (CD1) mice were purchased from the Laboratory Animal Center of the Institute of Genetics and Developmental Biology (Beijing, China). *Sgpl1* deletion mice of the C57/BL6 background were generated by using CRISPR/Cas9 gene editing technology (Fig. S3). In some experiments, female mice (21 days old) were injected with 5 IU equine chorionic gonadotropin 48 h before use to stimulate follicle development. The mice were raised in the Laboratory Animal Centre of China Agricultural University under controlled temperatures of 23 ± 2 °C with a 12/12 h light/dark cycle. All animal experimental procedures were approved by the Institutional Animal Care and Use Committee of China Agricultural University. Unless otherwise stated, the reagents were purchased from Sigma-Aldrich (St. Louis, MO, USA).

### In vitro culture of follicles and ovaries

Secondary preantral follicles (100–110 μm) were stripped from 12-day-old mice^7^. Then, follicles were cultured in 96-well plates supplemented with S1P (10–30 μM) and/or FSH (25 ng/ml) for 4 days, and the diameters of follicles were measured daily. The follicle culture medium was bicarbonate-buffered minimum Eagle medium-alpha with Earle balanced salts (Thermo Fisher Scientific, Waltham, MA, USA) supplemented with 1% ITS (I3146), 100 UI/ml penicillin-streptomycin, 0.23 mM pyruvate, and 3 mg/ml bovine serum albumin. For the analysis of calcium levels and the binding affinity of NPR2 for NPPC, preantral follicles were cultured without or with S1P (30 μM) for 2 h. For the analysis of the gene, protein, S1P, and cGMP levels, the ovaries were collected from 12- to 14-day-old WT and KO mice. In some experiments, ovaries isolated from 10-day-old mice were cultured without or with S1P (30 μM) for 4 days. For the BrdU assay, the ovaries were cultured with 1 μM 5-bromo-2′-deoxyuridine for 3 h. The ovaries were cultured in Dulbecco’s modified Eagle’s medium (DMEM)/F12 (Thermo Fisher Scientific) supplemented with ITS and penicillin-streptomycin. All cultures were carried out at 37 °C in an atmosphere of 5% CO_2_.

### Isolation and culture of Leydig cells

Leydig cells were isolated from 21- to 23-day-old mice according to a previous report with slight modification^36^. Briefly, the internal tissue of testis was digested with collagenase II (C6885). The resuspended cells were filtered and layered onto a 8 ml solution of 60, 37, 26, and 21% Percoll (P4937), and centrifuged at 3000 × *g* for 30 min at 4 °C. The Leydig cells were harvested at the interface between 60 and 37% Percoll, and the purity was identified by HSD3B immunofluorescence staining. The Leydig cells were cultured in DMEM/F12 medium supplemented with S1P, JTE-013, and/or VPC23019 (APExBIO, Houston, TX, USA) for the indicated time.

### Immunofluorescence and histological analysis

For immunofluorescence, the tissue samples were fixed in 4% paraformaldehyde (PFA) at 4 °C overnight, embedded in paraffin, and sectioned at 5 μm. The sections were dewaxed, rehydrated, and subjected to antigen retrieval. The Leydig cells were fixed with 4% PFA for 20 min and permeabilized with phosphate-buffered saline (PBS) solution containing 0.3% Triton X-100 (PBST) for 30 min. After blocking with 10% normal donkey serum, the sections or cells were incubated with primary antibodies (Table S1) and then incubated with Alexa Fluor 488- or 555-conjugated secondary antibodies (1:100, Thermo Fisher Scientific). Finally, the samples were counterstained with DAPI. Immunofluorescent staining was examined using a Nikon A1 laser scanning confocal microscope (Nikon, Tokyo, Japan). All the positive signal counts were completed by the blinded observer. For phenotypic analysis, the sections of ovaries were stained with periodic acid/Schiff reagent. The number of follicles of different types was counted by examining serial sections through the entire ovary^7^. The sections of testes were stained with hematoxylin and eosin.

### Western blotting

Total proteins were extracted in WIP buffer (Cell Chip Biotechnology, Beijing, China) with 1 mM phenylmethylsulfonyl fluoride on ice. 30 μg protein from each sample were separated by 10% SDS-polyacrylamide gel electrophoresis and then transferred to polyvinylidene fluoride membranes (Millipore, Billerica, MA, USA). After blocking with 5% nonfat milk, the membranes were incubated with primary antibodies (Table S1) overnight at 4 °C, and then incubated with horseradish peroxidase-conjugated secondary antibodies (each diluted 1:5000, ZSGB-BIO, Beijing, China). The blots were detected using the SuperSignal West Pico Kit and visualized by the Tanon 5200 chemiluminescent imaging system (Tanon, Shanghai, China). GAPDH or β-actin was used as a loading control.

### RNA extraction and analysis

Total RNA was extracted and purified by the RNeasy micro-RNA Isolation Kit (Qiagen, Valencia, CA, USA), and then reverse transcribed into cDNA by the QuantiTect Reverse Transcription System (Qiagen). qRT-PCR was conducted and analyzed on an ABI 7500 instrument (Applied Biosystems, CA, USA) using a standard protocol. For single-cell RNA-seq analysis, RNA was extracted from granulosa cells of 14-day-old WT and KO mice, and transcriptome analysis was performed by Annoroad Gene Technology Co., Ltd. (Beijing, China). For RNA-seq analysis, RNA was extracted from testes of 21-day-old WT and KO mice, and analyzed by Annoroad Gene Technology Co., Ltd. (Beijing, China). The RNA-seq data were partially verified by qRT-PCR and western blotting. The qRT-PCR primers are listed in Supplementary Table S2.

### TUNEL assay

Apoptotic cells were detected using the Click-iT Plus TUNEL Assay (1982275, Thermo Fisher Scientific). Briefly, the rehydrated sections or cultured cells were fixed with 4% PFA, permeabilized, and then incubated with a TUNEL reaction mixture. After washing with PBS, the smaples were counterstained with DAPI. The incorporated fluorescence was visualized under the confocal microscope.

### Measurement of the intracellular calcium levels and the binding affinity of NPR2 for NPPC

Preantral follicles were incubated in culture medium containing 5 µM Fluo 3-AM (Dojindo Laboratories, Kumamoto, Japan) for the detection of calcium levels, or incubated in culture medium containing 100 nM mono-5-(and 6)-carboxyfluorescein-labeled NPPC (FAM-NPPC; Phoenix Pharmaceuticals, Belmont, CA) for the detection of the binding affinity of NPR2 for NPPC as described previously^5^. The fluorescence was detected by the confocal microscope. All quantifications were performed using the Nikon NIS Elements BR 5.10 software. The fluorescence intensity represents the intracellular calcium levels (pseudocolor) and the binding affinity of NPR2 for NPPC (green), respectively.

### Measurement of cGMP levels in the ovaries

Ovarian tissues were solubilized in 200 µl of 5% trichloroacetic acid (TCA). After removing TCA, the samples were used for cGMP assay by a cGMP enzyme immunoassay kit obtained from Cayman Chemicals (Ann Arbor, MI, USA).

### Measurement of S1P levels in mouse gonads

The measurement of S1P was performed on liquid chromatography-tandem mass spectrometry (LC-MS/MS) with D-erythro-sphingosine-1-phosphate (S1P) as the standard and C17-D-erythro-sphingosine-1-phosphate (a 17-carbon analog of S1P, C17-S1P) as the internal standard. The method for sample processing is consistent with the previous report^37^. In total, 10 µl of the supernatant was injected into the LC-MS/MS system (LC, Shimazu Nexera X2 LC-30AD, Shimadzu, Kyoto, Japan; MS, AB SCIEX QTRAP 4500, AB SCIEX, MA, USA) equipped with an electrospray ionization source and multiple reaction monitoring (MRM) for analysis. The concentrations on the S1P standard curve were 0.1, 0.5, 1, 5, and 10 ng/ml. LC separation was performed on a Kinetex C18 column (2.1 × 100 mm, 2.6 μm) (Phenomenex, Torrance, CA, USA) at 35 °C. The mobile phase consisted of 0.1% formic acid-water (eluent A) and acetonitrile (eluent B) at a flow rate of 0.3 ml/min. The retention times of S1P and C17-S1P were 9.07 and 8.85 min, respectively. MS analysis was performed in positive ion mode with the following settings: curtain gas, 35 psi; ion spray voltage, 4500 V; and ion source heater temperature, 550 °C. According to the MRM, the ion transitions of S1P were 380.2 → 264.1 and those of C17-S1P were 366.1 → 250.1, with the same DP of 71 V and CE of 19 V.

### Statistical analysis

All experiments were independently performed at least three times with different mice or cell preparations. The qualitative data reported are representative results obtained in replicate experiments and presented as the mean ± SEM. Statistical analysis was performed using SPSS 20.0 (SPSS, Inc., Chicago, IL, USA). The differences among groups were determined using Student’s *t* test or one-way analysis of variance. Values of *p* < 0.05 were considered significant.

## Supplementary information

Supplementary Figure legends

Supplementary Tables

Figure S1

Figure S2

Figure S3

Figure S4

Figure S5

Figure S6

Figure S7

Figure S8

Figure S9

Figure S10

Figure S11

## Data Availability

RNA-seq data have been submitted to the NCBI Sequence Read Archive (SRA; https://submit.ncbi.nlm.nih.gov/subs/sra/) under accession number PRJNA723162 for female and PRJNA724022 for male. All data generated or analyzed during this study are available from the corresponding author on reasonable request.
