# Supplementary material for: Sgpl1 deletion elevates S1P levels, contributing to NPR2 inactivity and p21 expression that block germ cell development
Source: Cell Death Dis. 2021 Jun 3;12(6):574. doi: 10.1038/s41419-021-03848-9 (PMC8175456; doi:10.1038/s41419-021-03848-9)
Supplement: Supplementary file 1 — Supplementary Figure legends [file 41419_2021_3848_MOESM1_ESM.docx]

**Supplemental Material**

Feifei Yuan, et al.

***Sgpl1* deletion elevates S1P levels, contributing to NPR2 inactivity and p21 expression that block germ cell development**

**Supplementary Figure Legends**

**Fig. S1. Quantitative RT-PCR analysis of** ***Sgpl1*, *Sgpp1* and *Sgpp2* expression in granulosa cells and testes. a** Comparison of the mRNA levels of *Sgpl1*, *Sgpp1* and *Sgpp2* in granulosa cells isolated from 21-day-old mice. (*n* = 3 independent experiments). **b** Comparison of the mRNA levels of *Sgpl1*, *Sgpp1* and *Sgpp2* in testes isolated from 21-day-old mice (*n* = 3 independent experiments). Bars indicate the mean ± SEM. **P* < 0.05 and ****P* < 0.001.

**Fig. S2. Colocalization of SGPL1 and HSD3B in the testis.** Immunofluorescence double-labeling for SGPL1 (green) and HSD3B (red) in the testes isolated from 60-day-old mice. (*n* = 3 independent experiments). Scale bars: 100 μm.

**Fig. S3. Generation of *Sgpl1* KO mice by CRISPR/Cas9.** **a** A schematic diagram illustrating the gene targeting strategy for the generation of *Sgpl1* KO mice. Exon 3 was specifically removed in this strategy. **b** Information about the sgRNA in this target strategy. The target sequences were designed by using an online CRISPR design tool (<http://crispr.mit.edu>). **c** DNA graph represents PCR results of WT and mutant alleles. The *Sgpl1^+/+^* (WT) and *Spgl1^-/-^* (KO) mice used in this study were obtained by heterozygous mating (*Sgpl1^+/-^*× *Sgpl1^+/-^*). Genotyping for the *Sgpl1* mutation was performed using the primers 5’-CCT TAG CAC ACA GTA CAC TGC CAC AG-3’, 5’-ACC AAG CAC TGC CTG TAG GGA GA-3’ and 5’-AGG GCT CCA CCT CAG CAA TAA TCT T-3’ to amplify a 376 bp WT and/or a 221 bp mutant allele fragment.

**Fig. S4. The effect of *Sgpl1* deletion on cell proliferation and apoptosis in the ovary. a, b** Ki-67 staining (green) to detect cell proliferation (**a**) and TUNEL staining (green) to detect cell apoptosis (**b**) in ovaries from WT and KO mice. (*n* = 3 independent experiments). The nuclei were counterstained by DAPI (blue). Scale bars: 100 μm. **c** Quantitative analysis of granulosa cells positive for Ki-67 in WT and KO mice. The percentage of positive granulosa cells in secondary follicles was counted. (*n* = 3 independent experiments. The total number of 36 secondary follicles was scored in each group). **d** The mRNA levels of *caspase-3* in ovaries of WT and KO mice. (*n* = 3 independent experiments). **e** The protein levels of cleaved caspase-3 in ovaries of WT and KO mice. (*n* = 3 independent experiments). GAPDH was used as a loading control. Bars indicate the mean ± SEM. ns: no significance, ****P* < 0.001 vs. the WT group.

**Fig. S5. Quantitative RT-PCR analysis of *p21* expression in the ovary.** Comparison of the mRNA levels of *p21* in granulosa cells and oocytes isolated from 21-day-old mice. (*n* = 3 independent experiments). Bars indicate the mean ± SEM. ***P* < 0.01.

**Fig. S6. The effect of S1P on preantral follicle growth *in vitro*. a** Morphology of cultured preantral follicles at day 0 and day 4 without or with different concentrations of S1P (10, 20, 30 μM) and/or FSH (25 ng/ml). (*n* = 3 independent experiments. The total number of 36 secondary follicles was used in each group). Scale bars: 100 μm. **b** Histological analysis of 10-day-old ovaries treated with FSH and/or S1P (30 μM) for 4 days. The dashed line represents the diameter of the largest preantral follicle in each slice. (*n* = 3 independent experiments. The total number of 12 ovaries was used in each group). Scale bars: 100 μm. **c** Quantitative RT-PCR analysis of *inhibin-α* mRNA levels after treatment with FSH and/or S1P (30 μM) for 4 days. (*n* = 3 independent experiments). Treatment with S1P decreased the expression of inhibin-α, which indicated the low-quality of follicle development. Bars indicate the mean ± SEM. ns: no significance.

**Fig. S7. Purity of Leydig cell cultures.** Leydig cells were isolated from 21- to 23-day-old mice. The purity of Leydig cells was assessed by immunofluorescence staining with the Leydig cell marker HSD3B (red) and the purity of Leydig cells was approximately 90%. (*n* = 3 independent experiments). The nuclei were counterstained by DAPI (blue). Scale bars: 100 μm.

**Fig. S8. The effect of JTE-013 and VPC23019 on S1P-promoted *p21* mRNA expression in Leydig cells. a, b** Quantitative RT-PCR analysis of *p21* mRNA levels in Leydig cells after treatment with different concentrations of JTE-013 (**a**) or VPC23019 (**b**) for 6 hours. (*n* = 3 independent experiments). Bars indicate the mean ± SEM. ns: no significance, **P* < 0.05, ***P* < 0.01 and ****P* < 0.001 vs. the control group.

**Fig. S9. The changes in the expression of representative genes were validated by western blotting. a, b** The protein levels of CDK2 and inhibin-α in the ovaries of 14-day-old WT and KO mice. (*n* = 3 independent experiments). **c, d** The protein levels of inhibin-α, HSD3B and GLI1 in the testes of 21-day-old WT and KO mice. (*n* = 3 independent experiments). GAPDH was used as a loading control. Bars indicate the mean ± SEM. **P* < 0.05, ***P* < 0.01 and ****P* < 0.001 vs. the WT group.

**Fig. S10. Representative MRM chromatograms of S1P and internal standards in the ovary. a** The blank sample spiked with S1P (10 ng/mL) and internal standards C17-S1P (1 ng/mL). **b, c** Detection of S1P levels in ovarian samples from WT (**b**) and KO (**c**) mice spiked with internal standards C17-S1P (1 ng/mL).

**Fig. S11. Uncropped scans of the most important western blotting results**. **a** SGPL1 in the black dashed line box was used in Fig. 1c. **b, c** NPPC (**b**) and NPR2 (**c**) in the black dashed line box were used in Fig. 3b. **d, e** p21 (**d**) and PCNA (**e**) in the black dashed line box were used in Fig. 3j. **f** Cleaved caspase-3 in the black dashed line box was used in Fig. 5g. **g** CDK2 in the black dashed line box was used in Fig. S9a. **h-j** Inhibin-a (**h**), HSD3B (**i**) and GLI1 (**j**) in the black dashed line box were used in Fig. S9c.
