# Supplementary material for: Sgpl1 deletion elevates S1P levels, contributing to NPR2 inactivity and p21 expression that block germ cell development
Source: Cell Death Dis. 2021 Jun 3;12(6):574. doi: 10.1038/s41419-021-03848-9 (PMC8175456; doi:10.1038/s41419-021-03848-9)
Supplement: Supplementary file 2 — Supplementary Tables [file 41419_2021_3848_MOESM2_ESM.docx]

**Supplemental Material**

Feifei Yuan, et al.

***Sgpl1* deletion elevates S1P levels, contributing to NPR2 inactivity and p21 expression that block germ cell development**

**Supplementary Tables**

**Table S1: List of primary antibodies used in immune detection.**

| Antibody | Catalog Code | Source | Host | Dilution | |
| --- | --- | --- | --- | --- | --- |
|  |  |  |  | IF | WB |
| BrdU | ab1893 | Abcam | Sheep | 1:200 |  |
| Cleaved Caspase-3 | 9664 | Cell Signaling Technology | Rabbit | 1:50 | 1:1000 |
| HSD3B | sc515120 | Santa Cruz Biotechnology | Rabbit | 1:50 | 1:1000 |
| Ki-67 | 9129s | Cell Signaling Technology | Rabbit | 1:100 |  |
| p21 | ab188224 | Abcam | Rabbit | 1:50 | 1:1000 |
| PCNA | 2586 | Cell Signaling Technology | Mouse | 1:1000 | 1:1000 |
| SGPL1 | ab56183 | Abcam | Rabbit | 1:100 | 1:1000 |
| CDK2 | ab32147 | Abcam | Rabbit |  | 1:1000 |
| GLI1 | 3538 | Cell Signaling Technology | Rabbit |  | 1:1000 |
| inhibin-α | ab81322 | Abcam | Rabbit |  | 1:1000 |
| NPPC | sc374043 | Santa Cruz Biotechnology | Rabbit |  | 1:1000 |
| NPR2 | ab14357 | Abcam | Rabbit |  | 1:1000 |
| β-actin | ab8826 | Abcam | Mouse |  | 1:1000 |
| GAPDH | 5174 | Cell Signaling Technology | Rabbit |  | 1:1000 |

IF: Immunofluorescence; WB: Western blotting

**Table S2. Primers for qRT-PCR**

| **Genes** | **Forward primer (5’-3’)** | **Reverse primer (5’-3’)** |
| --- | --- | --- |
| *3βHSD* | GCTGCTGCACAGGAATAAAGG | CATGCCTGCTTCGTGACCAT |
| *Aldoa* | CGTGTGAATCCCTGCATTGG | CAGCCCCTGGGTAGTTGTC |
| *Bax* | TTTCATCCAGGATCGAGCAGG | GCAAAGTAGAAGAGGGCAACCAC |
| *Bcl-2* | CTACCGTCGTGACTTCGCA | TACCCAGCCTCCGTTATCC |
| *Caspase-3* | AGAGGAATGATTGGGGGTG | TTGCTAGGCAGTGGTAGCG |
| *Caspase-9* | CGGAATCACCAATCATTACAT | AGAAACGCCCACAACTGC |
| *Ccna1* | GCCCGACGTGGATGAGTTT | AGGAGGAATTGGTTGGTGGTT |
| *Ccnd2* | GAGTGGGAACTGGTAGTGTTG | CGCACAGAGCGATGAAGGT |
| *Cdk2* | CCTGCTTATCAATGCAGAGGG | TGCGGGTCACCATTTCAGC |
| *Cdk4* | AAGGTCACCCTAGTGTTTGAGC | CCGCTTAGAAACTGACGCATTAG |
| *Cyp11a1* | CTGGCGACAATGGTTGGCTA | GCCCAGCTTCTCCCTGTAAA |
| *Cyp17a1* | TGACCAGTATGTAGGCTTCAGTCG | TCCTTCGGGATGGCAAACTCTC |
| *Esr1* | CCTCCCGCCTTCTACAGGT | CACACGGCACAGTAGCGAG |
| *Esr2* | CTGTGCCTCTTCTCACAAGGA | TGCTCCAAGGGTAGGATGGAC |
| *Gli1* | ATCACCTGTTGGGGATGCTG | TCCGACAGCCTTCAAACGTG |
| *Gtsf1* | CTTCTGGACTGAGCACTTGA | ATGTGCTCTCAGCCAGAGTC |
| *Igf1* | CTGGACCAGAGACCCTTTGC | GGACGGGGACTTCTGAGTCTT |
| *Inhibin-α* | CCTTTTGCTGTTGACCCTACG | AGGCATCTAGGAATAGAGCCTTC |
| *Insl6* | AAGATTCACAAACCCAGTCCCT | ATCAACAAAGGGGAGGCACG |
| *Kif2a* | CGATGGCCGAATACACCAAG | CGTGCTGAACCAACCACTCT |
| *Kif2c* | AATCAAGAAGTGCTCAGGGGG | TTTGCGTTTTTGCTTCGGGA |
| *Ldha* | TTCAGCGCGGTTCCGTTAC | CCGGCAACATTCACACCAC |
| *Nppc* | GGTCTGGGATGTTAGTGCAGCTA | TAAAAGCCACATTGCGTTGGA |
| *Npr2* | GCTGACCCGGCAAGTTCTGT | ACAATACTCGGTGACAATGCAGAT |
| *p21* | CCTGGTGATGTCCGACCTG | CCATGAGCGCATCGCAATC |
| *Patz1* | CCTATGGCAACAAAGAAGGCCA | TGGGTACTTCTTCTCCCCGT |
| *Pcna* | TTGCACGTATATGCCGAGACC | GGTGAACAGGCTCATTCATCTCT |
| *S1pr1* | ATGGTGTCCACTAGCATCCC | CGATGTTCAACTTGCCTGTGTAG |
| *S1pr2* | ACAGCAAGTTCCACTCAGCAA | CTGCACGGGAGTTAAGGACAG |
| *S1pr3* | ACTCTCCGGGAACATTACGAT | CCAAGACGATGAAGCTACAGG |
| *S1pr4* | GTCAGGGACTCGTACCTTCCA | GATGCAGCCATACACACGG |
| *S1pr5* | TGTGTGTGCCTTCATTGTGC | CAGTAGGATGTTGGTGGCGTA |
| *Sgpl1* | TTTCCTCATGGTGTGATGGA | CCCCAGACAAGCATCCAC |
| *Sgpp1* | TACGGGCTGATTCTCATTCCC | GGTCCACCAATGGGTAGAAGA |
| *Sgpp2* | TTCACCCACTGGAATATCGACC | AAGTCTCACAACGGGAGGAAA |
| *Star* | ATGTTCCTCGCTACGTTCAAG | CCCAGTGCTCTCCAGTTGAG |
| *Tuba4a* | GGAGGGGACGACTCCTTCA | TGGGCCATTTCGGATCTCATC |
| *Rpl19* | CTGAAGGTCAAAGGGAATGTGTTC | TGGTCAGCCAGGAGCTTCTTG |

**Table S3. List of significantly changed transcripts in granulosa cells of *Sgpl1* KO mice identified by RNA-seq analysis.**

| **Genes** | **Description** | **Log2 Fold Change** |
| --- | --- | --- |
| *Scd1* | Acyl-CoA desaturase 1 | 4.668950957 |
| *Ccna1* | Cyclin-A1 | 4.481323953 |
| *Gm45033* | Predicted gene, 45033  LINE-1 retrotransposable element ORF2 protein | 3.838451682 |
| *Cadm4* | Cell adhesion molecule 4 | 3.695727145 |
| *Ppfia3* | Liprin-alpha-3 | 3.664623617 |
| *Comp* | Cartilage oligomeric matrix protein | 3.446356233 |
| *Gm12421* | Predicted gene, 12421  40S ribosomal protein S6 | 3.39649669 |
| *Cyp26b1* | Cytochrome P450 26B1 | 3.38671874 |
| *Vmn2r11* | Vomeronasal type-2 receptor 116 | 3.325984898 |
| *Palm2* | Paralemmin-2 | 3.295132368 |
| *Aldob* | Fructose-bisphosphate aldolase B | 3.172399492 |
| *Elovl6* | Elongation of very long chain fatty acids protein 6 | 2.747377125 |
| *Ctgf* | Connective tissue growth factor | 2.556055051 |
| *Retn* | Resistin | 2.332396042 |
| *Cdkn1c* | Cyclin-dependent kinase inhibitor 1C | 2.159395859 |
| *Ace* | Angiotensin-converting enzyme | 2.125683668 |
| *Wnt4* | Protein Wnt-4 | 2.124042422 |
| *Taf15* | TATA-binding protein-associated factor 2N | 2.080714513 |
| *Cdkn1a* | Cyclin-dependent kinase inhibitor 1 | 1.951856565 |
| *Msmo1* | Methylsterol monooxygenase 1 | 1.935087924 |
| *Mvk* | Mevalonate kinase | 1.761752723 |
| *Cyp51* | Lanosterol 14-alpha demethylase | 1.717512032 |
| *Numbl* | Numb-like protein | 1.66867236 |
| *Ebp* | 3-beta-hydroxysteroid-Delta (8), Delta (7)-isomerase | 1.561177261 |
| *Penk* | Proenkephalin-A | 1.560122729 |
| *Cd34* | Hematopoietic progenitor cell antigen CD34 | 1.530911477 |
| *B4galt1* | Beta-1,4-galactosyltransferase 1 | 1.469042266 |
| *Cdkn1b* | Cyclin-dependent kinase inhibitor 1B | 1.451832657 |
| *Hbegf* | Proheparin-binding EGF-like growth factor | 1.431516277 |
| *Cd74* | H-2 class II histocompatibility antigen gamma chain | 1.409690866 |
| *Vcl* | Vinculin | 1.408432737 |
| *Col8a1* | Collagen alpha-1(VIII) chain | 1.40459786 |
| *Impdh1* | Inosine-5'-monophosphate dehydrogenase 1 | 1.369804422 |
| *Hacd4* | Very-long-chain (3R)-3-hydroxyacyl-CoA dehydratase 4 | 1.301203185 |
| *Prkaca* | cAMP-dependent protein kinase catalytic subunit alpha | 1.280182651 |
| *Sqle* | Squalene monooxygenase | 1.237687507 |
| *Scd2* | Acyl-CoA desaturase 2 | 1.172642409 |
| *Ncor2* | Nuclear receptor corepressor 2 | 1.147935913 |
| *Jarid2* | Protein Jumonji | 1.05313379 |
| *Fam83d* | Protein FAM83D | -1.039393253 |
| *Csnk1d* | Casein kinase I isoform delta | -1.039622054 |
| *Nans* | Sialic acid synthase | -1.042238003 |
| *Cyp11a1* | Cholesterol side-chain cleavage enzyme, mitochondrial | -1.047027434 |
| *Cdk2* | Cyclin-dependent kinase 2 | -1.063832886 |
| *Igf1* | Insulin-like growth factor I | -1.079851377 |
| *Gne* | Bifunctional UDP-N-acetylglucosamine 2-epimerase/N-acetylmannosamine kinase | -1.106009452 |
| *Pfkl* | ATP-dependent 6-phosphofructokinase, liver type | -1.158462572 |
| *Bpgm* | Bisphosphoglycerate mutase | -1.162100638 |
| *Gmds* | GDP-mannose 4,6 dehydratase | -1.164975412 |
| *Ehf* | ETS homologous factor | -1.19082744 |
| *Pla2g4c* | Cytosolic phospholipase A2 gamma | -1.222172858 |
| *Aldoa* | Fructose-bisphosphate aldolase A | -1.225794282 |
| *Npl* | N-acetylneuraminate lyase | -1.354182009 |
| *Tacc3* | Transforming acidic coiled-coil-containing protein 3 | -1.358259451 |
| *Kdm1b* | Lysine-specific histone demethylase 1B | -1.373904522 |
| *Tpi1* | Triosephosphate isomerase | -1.41687682 |
| *Fbp1* | Fructose-1,6-bisphosphatase 1 | -1.421201095 |
| *Pkm* | Pyruvate kinase PKM | -1.498915346 |
| *Pecr* | Peroxisomal trans-2-enoyl-CoA reductase | -1.503024701 |
| *Ccnd2* | G1/S-specific cyclin-D2 | -1.542380307 |
| *Trp53i13* | Tumor protein p53-inducible protein 13 | -1.546582043 |
| *Mcm6* | DNA replication licensing factor MCM6 | -1.617981973 |
| *Hsd17b2* | Estradiol 17-beta-dehydrogenase 2 | -1.660586672 |
| *Acaa1a* | 3-ketoacyl-CoA thiolase A, peroxisomal | -1.66543686 |
| *Mcm3* | DNA replication licensing factor MCM3 | -1.681590368 |
| *Adcy3* | Adenylate cyclase type 3 | -1.688601048 |
| *Slc38a3* | Sodium-coupled neutral amino acid transporter 3 | -1.751824657 |
| *Gmppb* | Mannose-1-phosphate guanyltransferase beta | -1.76660356 |
| *Cpt2* | Carnitine O-palmitoyltransferase 2, mitochondrial | -1.77219088 |
| *Mecr* | Trans-2-enoyl-CoA reductase, mitochondrial | -1.789714565 |
| *Pgm2* | Phosphoglucomutase-1 | -1.811383066 |
| *Pgm1* | Phosphoglucomutase-2 | -1.819448527 |
| *Renbp* | N-acylglucosamine 2-epimerase | -1.827161879 |
| *Amdhd2* | N-acetylglucosamine-6-phosphate deacetylase | -1.924049622 |
| *Star* | Steroidogenic acute regulatory protein, mitochondrial | -1.975222274 |
| *Hk1* | Hexokinase-1 | -1.978107665 |
| *Scarb1* | Scavenger receptor class B member 1 | -2.163811479 |
| *Ccne1* | G1/S-specific cyclin-E1 | -2.187238749 |
| *Hk2* | Hexokinase-2 | -2.207068137 |
| *Acaa2* | 3-ketoacyl-CoA thiolase, mitochondrial | -2.207557499 |
| *Acads* | Short-chain specific acyl-CoA dehydrogenase, mitochondrial | -2.214062537 |
| *Gfpt2* | Glutamine--fructose-6-phosphate aminotransferase [isomerizing] 2 | -2.264825212 |
| *Ldha* | L-lactate dehydrogenase A chain | -2.39305026 |
| *Uap1l1* | UDP-N-acetylhexosamine pyrophosphorylase-like protein 1 | -2.438622795 |
| *Tuba1b* | Tubulin alpha-1B chain | -2.649487093 |
| *E2f4* | Transcription factor E2F4 | -2.834278503 |
| *Tuba4a* | Tubulin alpha-4A chain | -2.884998261 |
| *Galk1* | Galactokinase | -2.898054414 |
| *Inha* | Inhibin alpha chain | -2.916888713 |
| *Nppc* | C-type natriuretic peptide | -2.942928171 |
| *Gnas* | Guanine nucleotide-binding protein G(s) subunit alpha | -3.148032667 |
| *Nr4a3* | Nuclear receptor subfamily 4 group A member 3 | -3.276991147 |
| *E2f1* | Transcription factor E2F1 | -3.332457238 |
| *Pla2g4e* | Cytosolic phospholipase A2 epsilon | -4.080918471 |
| *Tuba3a* | Tubulin alpha-3 chain | -4.881246126 |
| *Acadvl* | Very long-chain specific acyl-CoA dehydrogenase, mitochondrial | -6.046153053 |

**Table S4. List of significantly changed transcripts in testes of *Sgpl1* KO mice identified by RNA-seq analysis.**

| **Genes** | **Description** | **Log2 Fold Change** |
| --- | --- | --- |
| *Gpx5* | Epididymal secretory glutathione peroxidase | 5.118867 |
| *Bcan* | Brevican core protein | 3.585735 |
| *Slc1a6* | Excitatory amino acid transporter 4 | 2.503519 |
| *Ier3* | Radiation-inducible immediate-early gene IEX-1 | 2.318205 |
| *Rin2* | Ras and Rab interactor 2 | 2.219976 |
| *Sept1* | Septin-1 | 1.932097 |
| *Ccnd2* | G1/S-specific cyclin-D2 | 1.818335 |
| *Slfn4* | Schlafen family member 12-like | 1.63386 |
| *Irf9* | Interferon regulatory factor 9 | 1.626001 |
| *Mmp11* | Stromelysin-3 | 1.547021 |
| *Mef2c* | Myocyte-specific enhancer factor 2C | 1.529529 |
| *Rasa4* | Ras GTPase-activating protein 4 | 1.504549 |
| *Sertad4* | SERTA domain-containing protein 4 | 1.498481 |
| *Zim1* | Zinc finger protein 354C | 1.490572 |
| *Epdr1* | Mammalian ependymin-related protein 1 | 1.400962 |
| *Rab3b* | Ras-related protein Rab-3B | 1.369117 |
| *Hspb1* | Heat shock protein beta-1 | 1.347103 |
| *Fshr* | Follicle-stimulating hormone receptor | 1.296214 |
| *Mapk13* | Mitogen-activated protein kinase 13 | 1.293969 |
| *Nfix* | Nuclear factor 1 X-type | 1.280805 |
| *Stra8* | Stimulated by retinoic acid gene 8 protein | 1.272822 |
| *Ndrg1* | Protein NDRG1 | 1.271773 |
| *Ndrg1* | Protein NDRG1 | 1.271773 |
| *Oas1c* | 2'-5'-oligoadenylate synthase 1A | 1.269574 |
| *Hlf* | Hepatic leukemia factor | 1.260334 |
| *Bcl2* | Apoptosis regulator Bcl-2 | 1.258000 |
| *Mlxipl* | Carbohydrate-responsive element-binding protein | 1.219613 |
| *Gm38393* | Predicted gene,  38393SNRPN upstream reading frame protein | 1.219006 |
| *Cdh1* | Cadherin-1 | 1.207864 |
| *Arhgef40* | Rho guanine nucleotide exchange factor 40 | 1.172937 |
| *Mid2* | Probable E3 ubiquitin-protein ligase MID2 | 1.156116 |
| *Dnase2a* | Deoxyribonuclease-2-alpha | 1.136475 |
| *Zfp40* | Zinc finger protein 182 | 1.127012 |
| *Fbln1* | Fibulin-1 | 1.111531 |
| *Acvrl1* | Serine/threonine-protein kinase receptor R3 | 1.089199 |
| *Ddr1* | Epithelial discoidin domain-containing receptor 1 | 1.087158 |
| *Dag1* | Dystroglycan | 1.086392 |
| *Trp53* | Cellular tumor antigen p53 | 1.081872 |
| *Amh* | Muellerian-inhibiting factor | 1.053521 |
| *Stat6* | Signal transducer and transcription activator 6 | 1.033516 |
| *Lama1* | Laminin subunit alpha-1 | 1.026766 |
| *Homer3* | Homer protein homolog 3 | 1.016305 |
| *Prkd1* | Serine/threonine-protein kinase D1 | 1.015007 |
| *Top1mt* | DNA topoisomerase I, mitochondrial | 1.014563 |
| *Prelp* | Prolargin | 1.008433 |
| *Kif2a* | Kinesin-like protein KIF2A | -1.01627 |
| *Dgcr6* | Protein DGCR6 | -1.06294 |
| *Kif18b* | Kinesin-like protein KIF18B | -1.06441 |
| *Aven* | Cell death regulator Aven | -1.15874 |
| *Col1a1* | Collagen alpha-1(I) chain | -1.16674 |
| *Napsa* | Napsin-A | -1.19444 |
| *Dhrs1* | Dehydrogenase/reductase SDR family member 1 | -1.21233 |
| *Mea1* | Male-enhanced antigen 1 | -1.21478 |
| *Irx2* | Iroquois-class homeodomain protein IRX-2 | -1.24377 |
| *Cd36* | Platelet glycoprotein 4 | -1.25191 |
| *Txnrd3* | Thioredoxin reductase 3 | -1.26064 |
| *Gstm5* | Glutathione S-transferase Mu 5 | -1.28926 |
| *Pla1a* | Phospholipase A1 member A | -1.29825 |
| *Dyrk1b* | Dual specificity tyrosine-phosphorylation-regulated kinase 1B | -1.30916 |
| *Chaf1a* | Chromatin assembly factor 1 subunit A | -1.30981 |
| *Cdkl3* | Cyclin-dependent kinase-like 3 | -1.32274 |
| *Hapln2* | Hyaluronan and proteoglycan link protein 2 | -1.37154 |
| *Zpbp* | Zona pellucida-binding protein 1 | -1.37576 |
| *Folh1* | Glutamate carboxypeptidase 2 | -1.37683 |
| *Slc22a18* | Solute carrier family 22 member 18 | -1.42226 |
| *Lipe* | Hormone-sensitive lipase | -1.42469 |
| *Cep128* | Centrosomal protein of 128 kDa | -1.44937 |
| *Spa17* | Sperm surface protein Sp17 | -1.46245 |
| *Ccdc113* | Coiled-coil domain-containing protein 113 | -1.48937 |
| *Grin2d* | Glutamate receptor ionotropic, NMDA 2D | -1.50884 |
| *Lhcgr* | Lutropin-choriogonadotropic hormone receptor | -1.56184 |
| *Kif9* | Kinesin-like protein KIF9 | -1.62533 |
| *Wnt3* | Proto-oncogene Wnt-3 | -1.65928 |
| *Tekt1* | Tektin-1 | -1.68595 |
| *Ccdc65* | Coiled-coil domain-containing protein 65 | -1.71706 |
| *Atp8b3* | Phospholipid-transporting ATPase IK | -1.8078 |
| *Igf1* | Insulin-like growth factor I | -1.86571 |
| *Klf4* | Krueppel-like factor 4 | -1.93867 |
| *Dnmt3l* | DNA (cytosine-5)-methyltransferase 3-like | -2.0906 |
| *Ncan* | Neurocan core protein | -2.14357 |
| *Marveld3* | MARVEL domain-containing protein 3 | -2.45765 |
| *Spaca1* | Sperm acrosome membrane-associated protein 1 | -2.66705 |
| *Npas1* | Neuronal PAS domain-containing protein 1 | -2.93087 |
| *Ppm1j* | Protein phosphatase 1J | -3.23449 |
| *Tssk3* | Testis-specific serine/threonine-protein kinase 3 | -3.44854 |
| *Vmn2r88* | Vomeronasal type-2 receptor 116 | -3.93892 |
| *Nmrk2* | Nicotinamide riboside kinase 2 | -4.55363 |
| *Prm2* | Protamine-2 | -5.57012 |
| *Hsd3b6* | 3 beta-hydroxysteroid dehydrogenase/Delta 5-->4-isomerase type 6 | -6.00279 |
| *Cyp11a1* | Cholesterol side-chain cleavage enzyme, mitochondrial | -6.1917 |
| *Cyp17a1* | Steroid 17-alpha-hydroxylase/17,20 lyase | -7.1319 |
| *Kif2b* | Kinesin-like protein KIF2B | -8.65786 |
