# Supplementary figures and images for: Sgpl1 deletion elevates S1P levels, contributing to NPR2 inactivity and p21 expression that block germ cell development
Source: Cell Death Dis. 2021 Jun 3;12(6):574. doi: 10.1038/s41419-021-03848-9 (PMC8175456; doi:10.1038/s41419-021-03848-9)

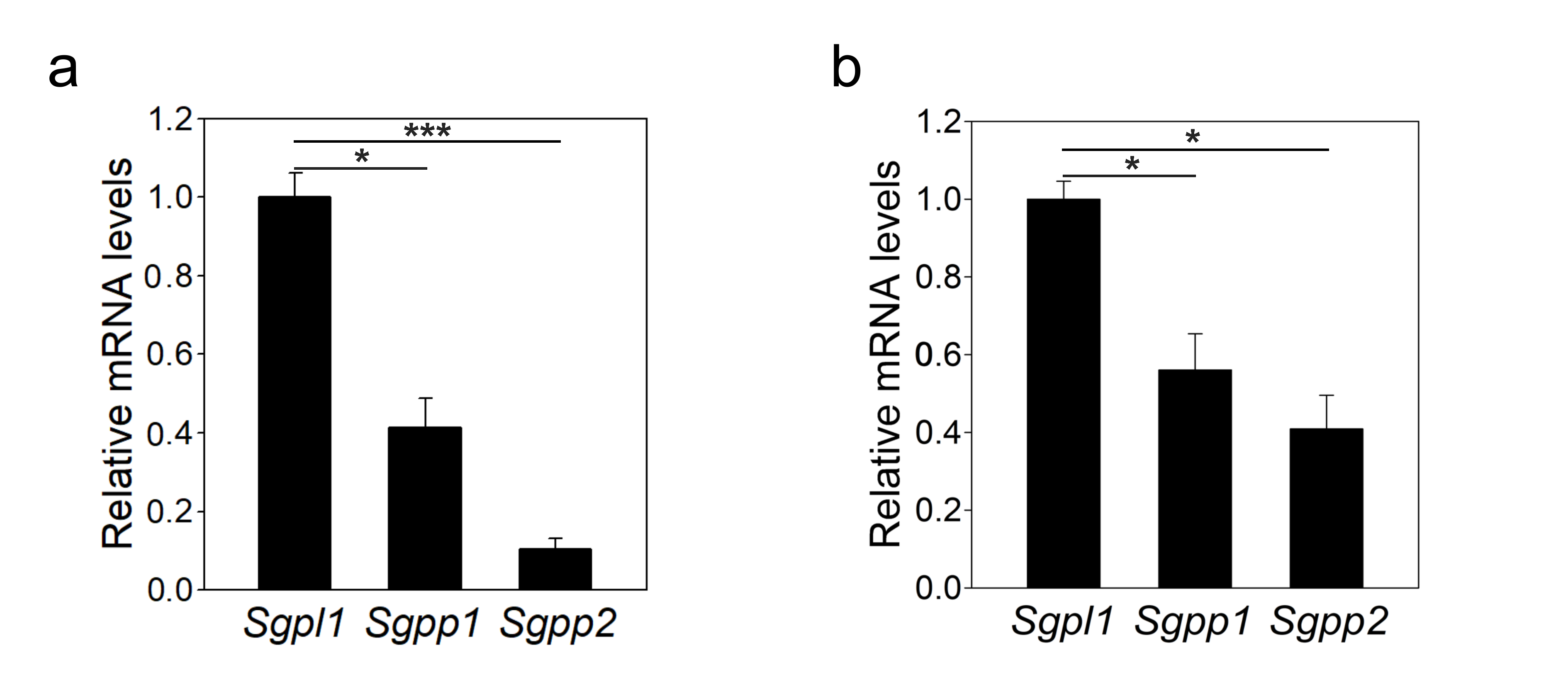

Supplement: Supplementary file 3 — Figure S1 [file 41419_2021_3848_MOESM3_ESM.tif]

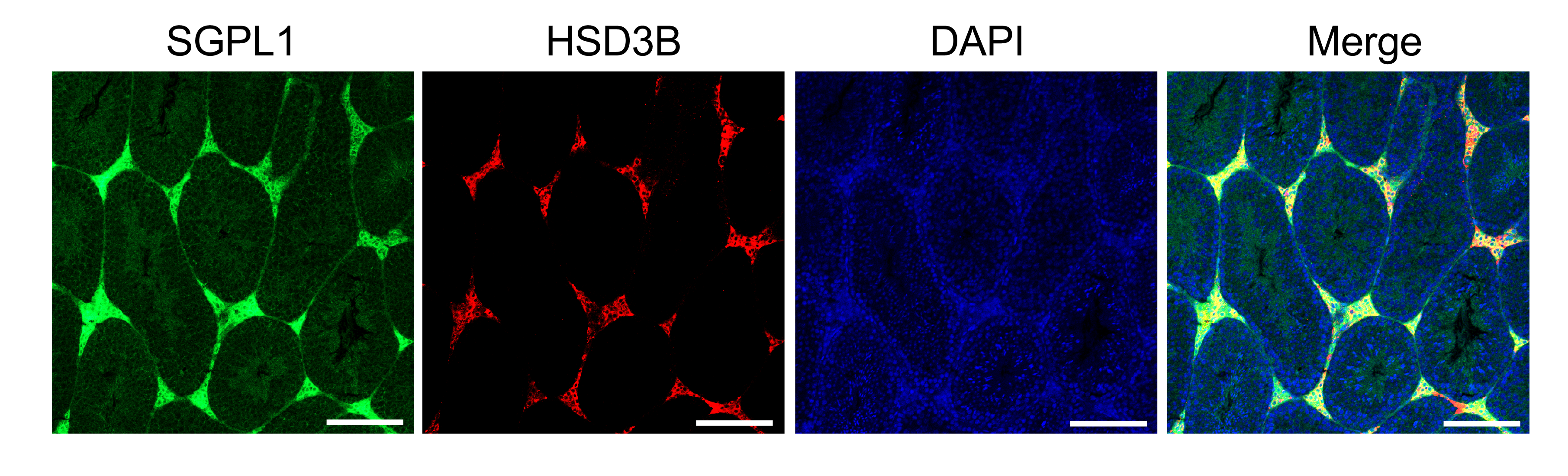

Supplement: Supplementary file 4 — Figure S2 [file 41419_2021_3848_MOESM4_ESM.tif]

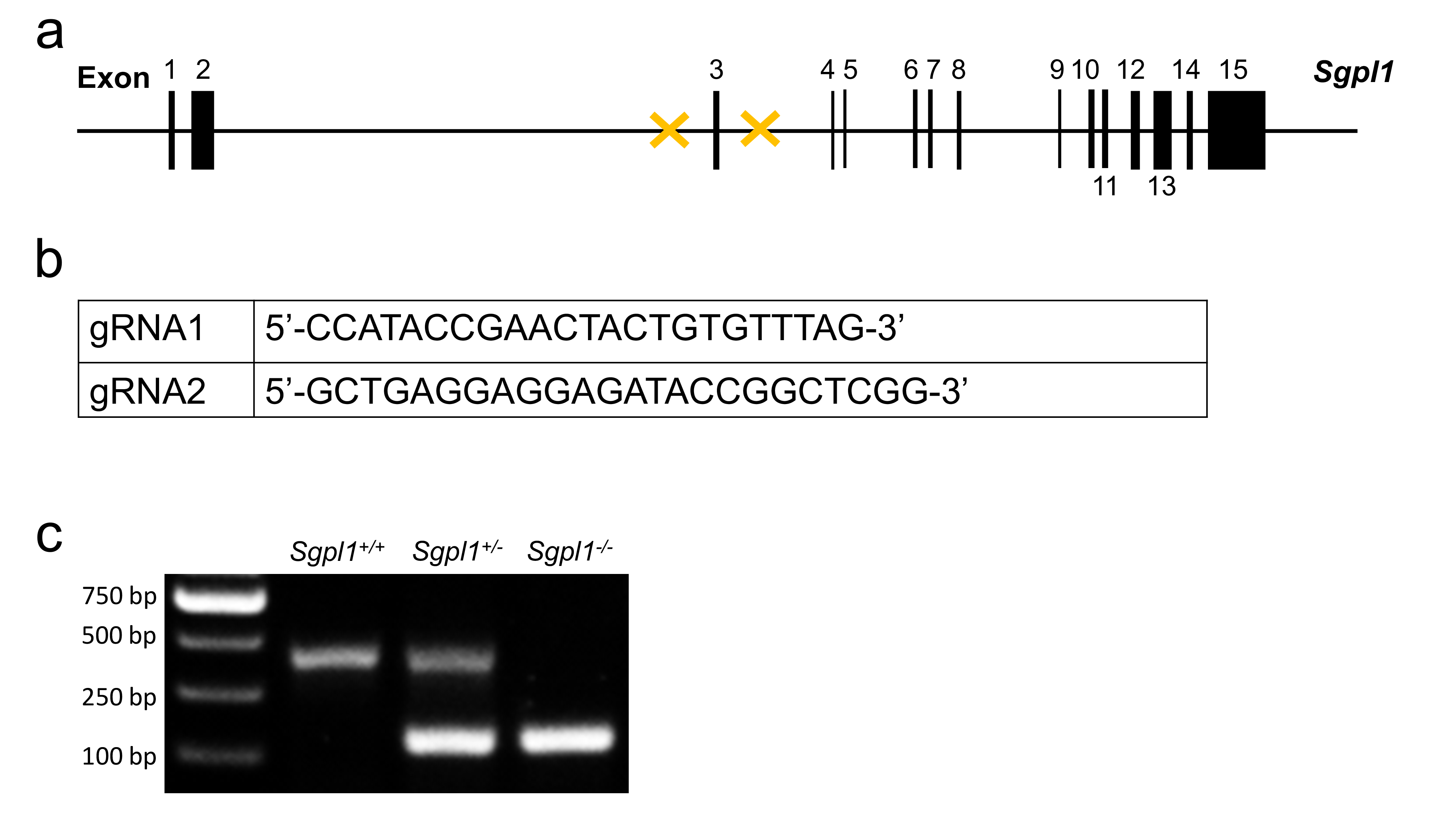

Supplement: Supplementary file 5 — Figure S3 [file 41419_2021_3848_MOESM5_ESM.tif]

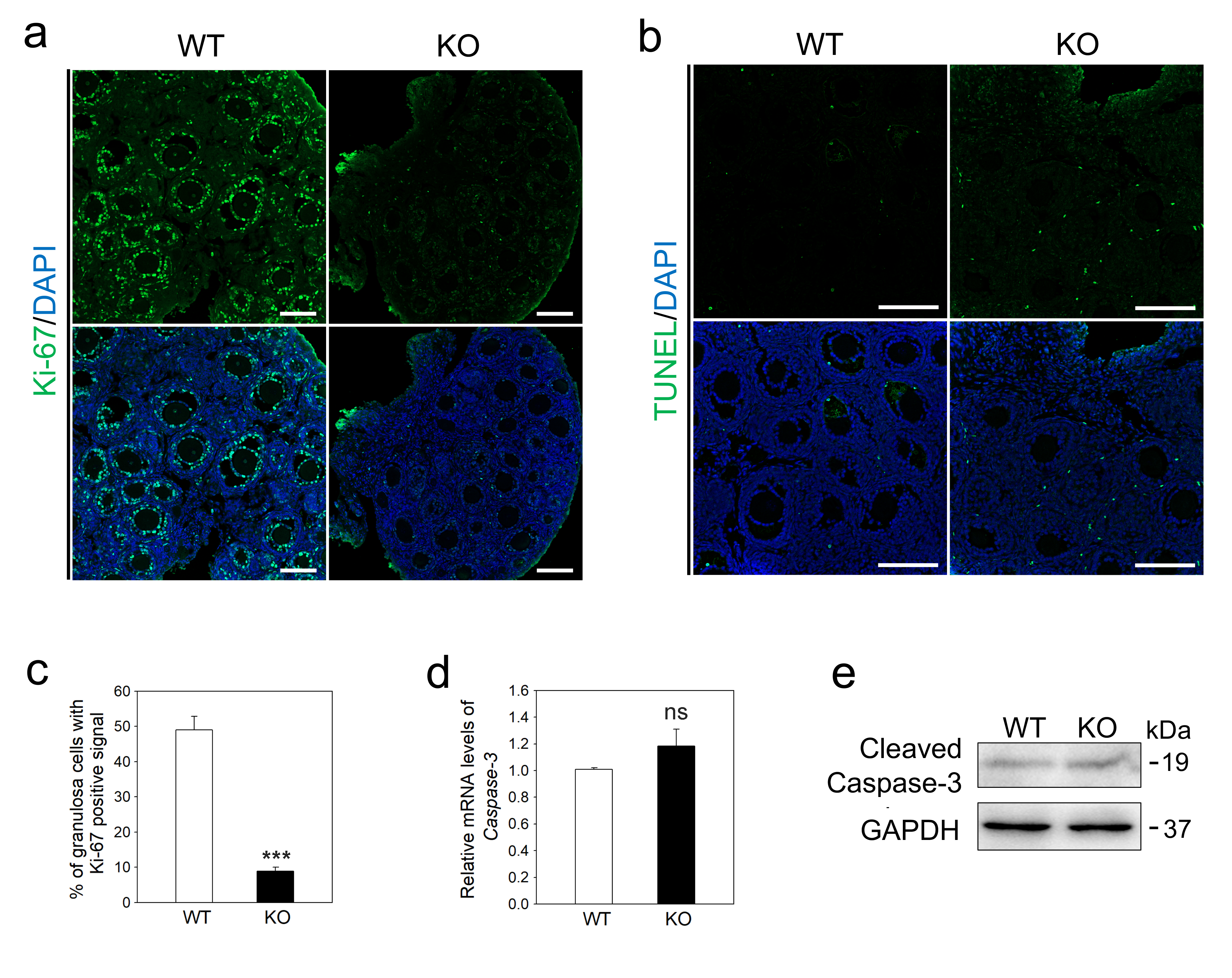

Supplement: Supplementary file 6 — Figure S4 [file 41419_2021_3848_MOESM6_ESM.tif]

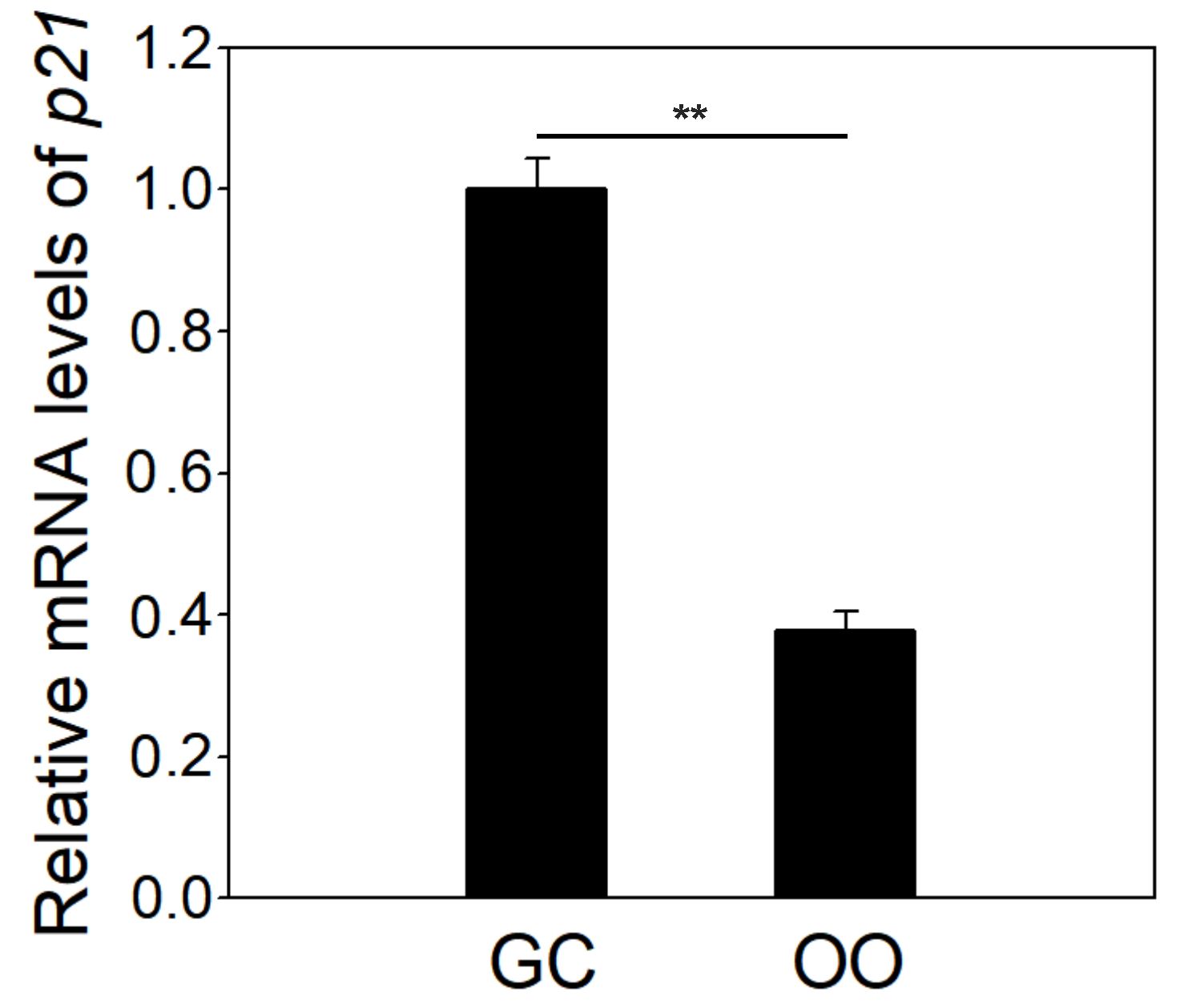

Supplement: Supplementary file 7 — Figure S5 [file 41419_2021_3848_MOESM7_ESM.tif]

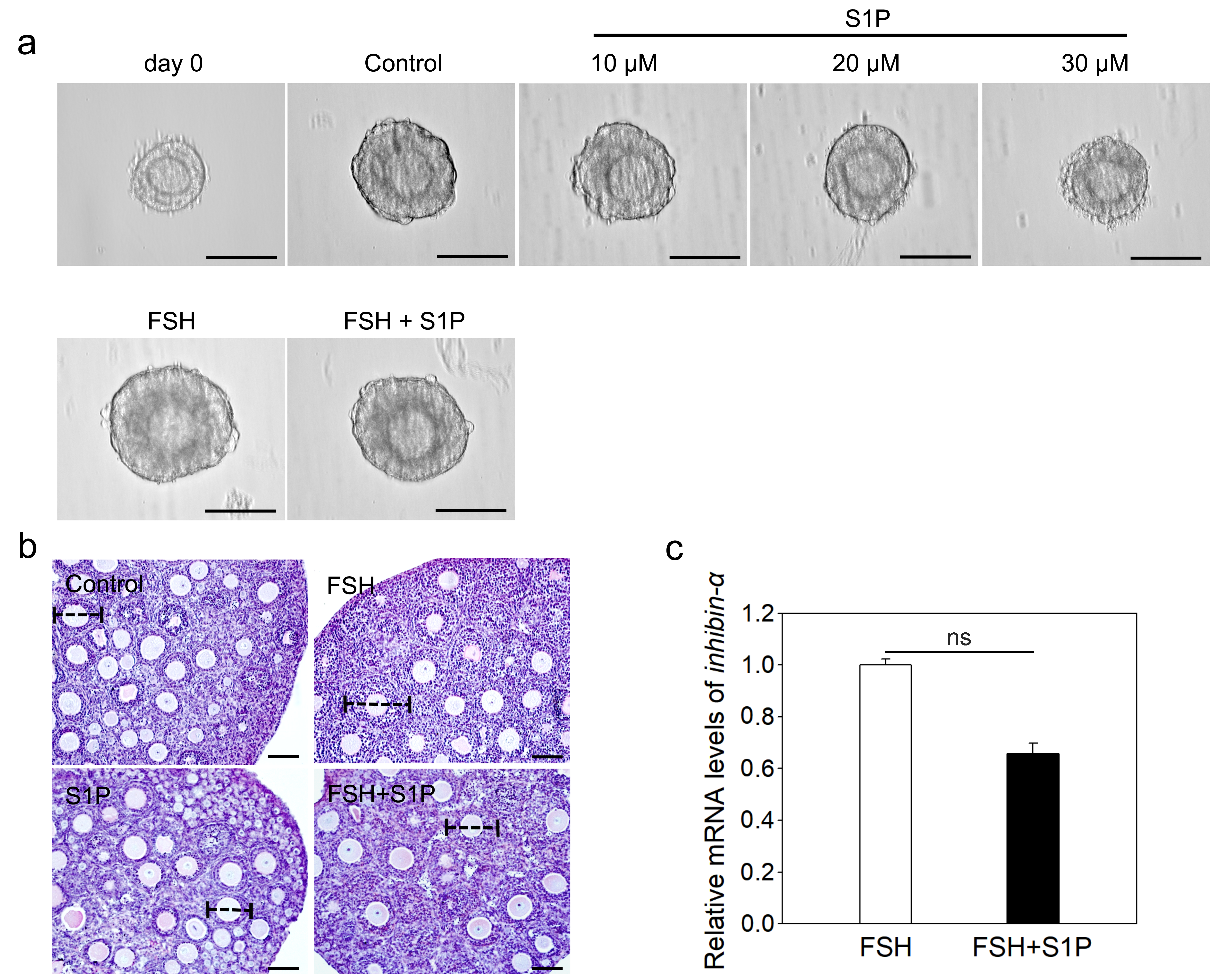

Supplement: Supplementary file 8 — Figure S6 [file 41419_2021_3848_MOESM8_ESM.tif]

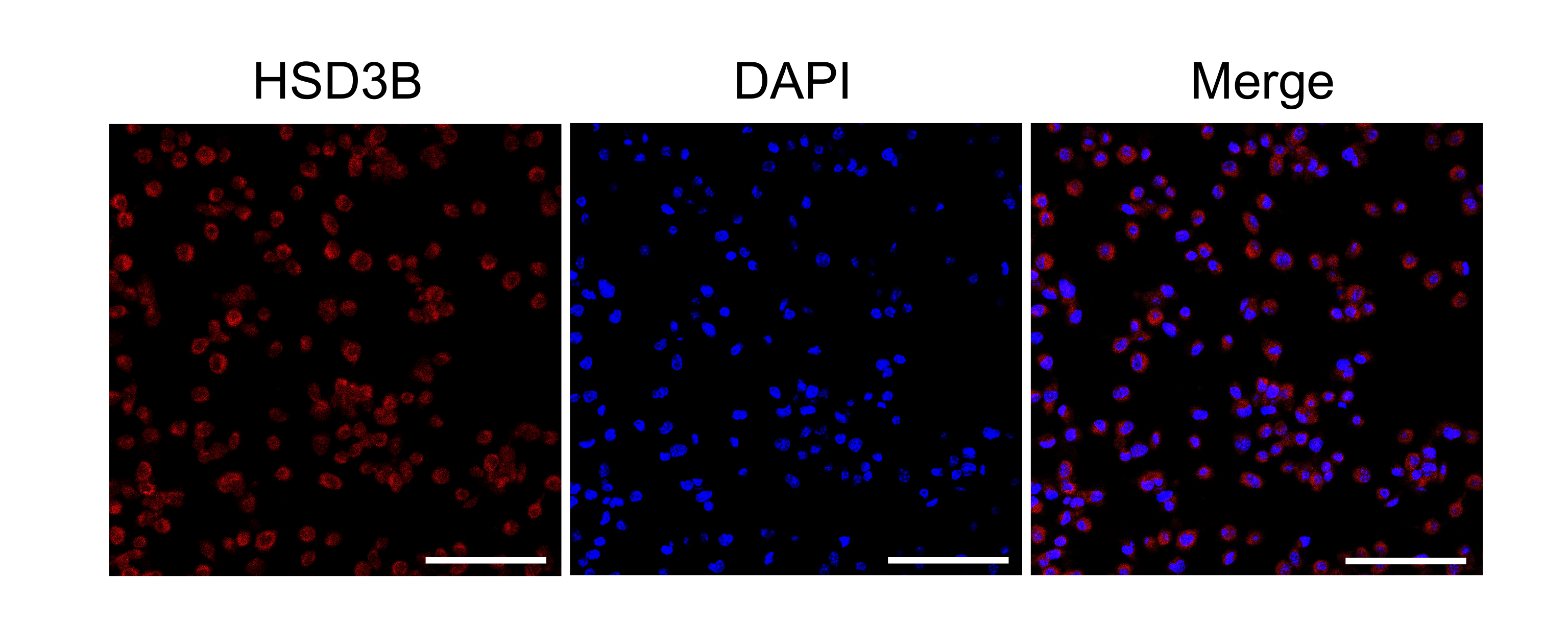

Supplement: Supplementary file 9 — Figure S7 [file 41419_2021_3848_MOESM9_ESM.tif]

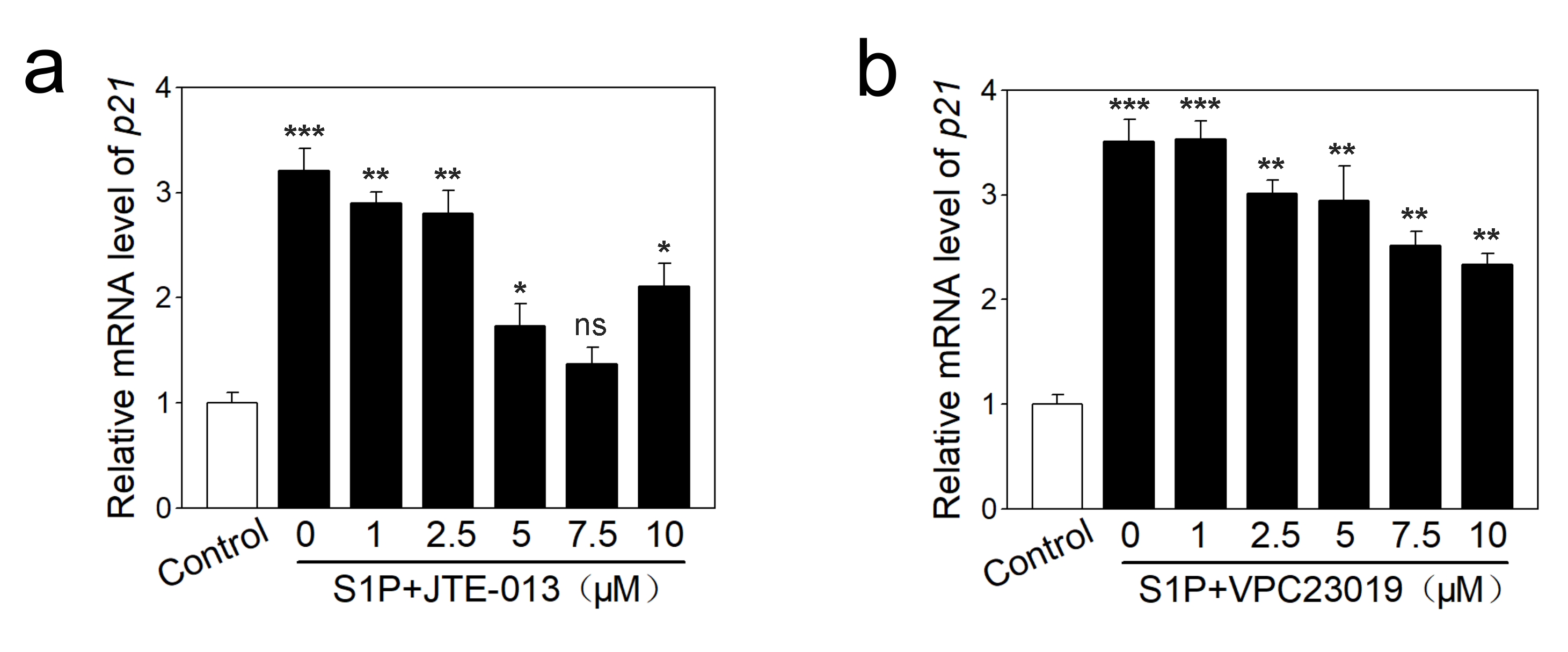

Supplement: Supplementary file 10 — Figure S8 [file 41419_2021_3848_MOESM10_ESM.tif]

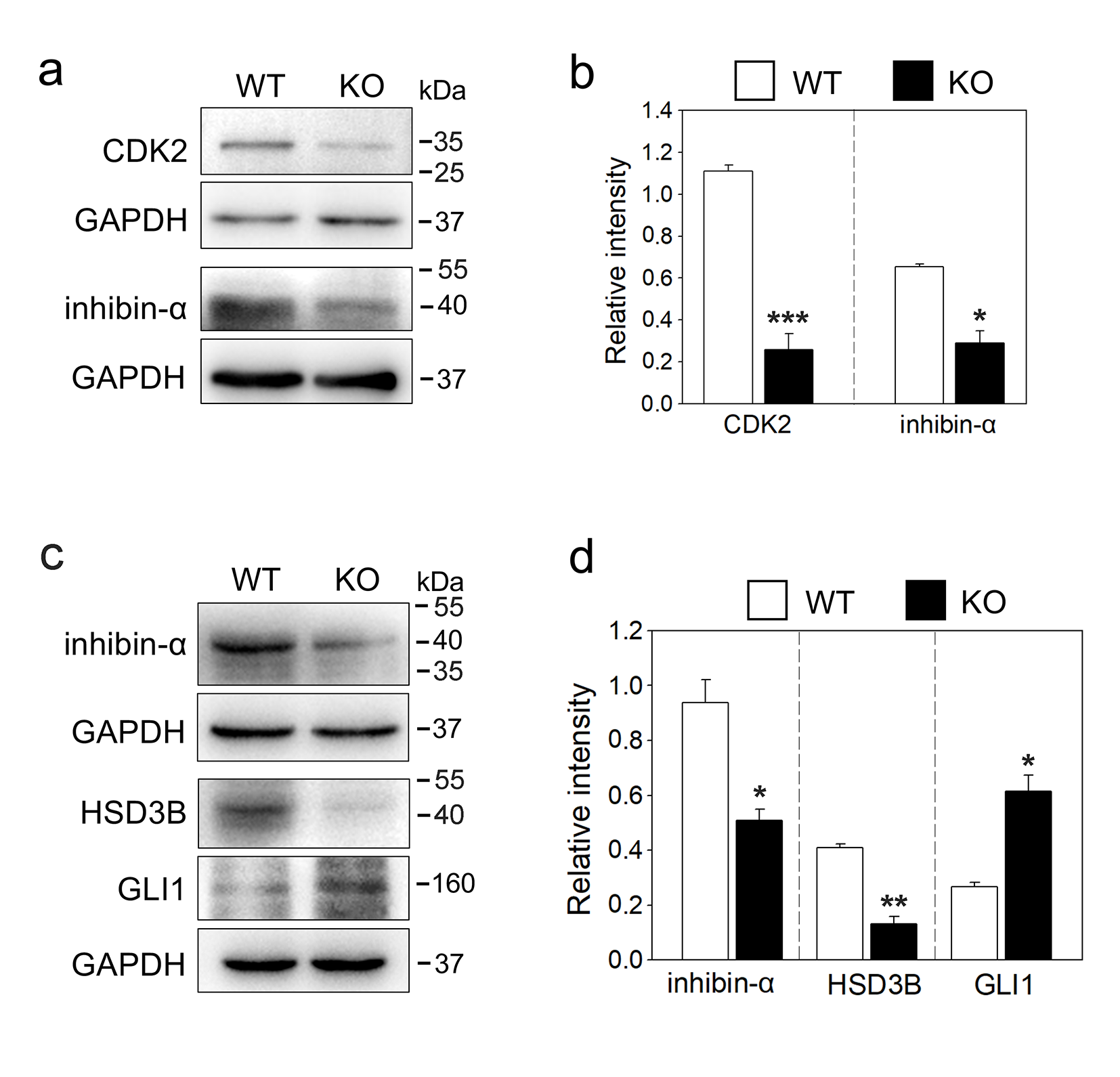

Supplement: Supplementary file 11 — Figure S9 [file 41419_2021_3848_MOESM11_ESM.tif]

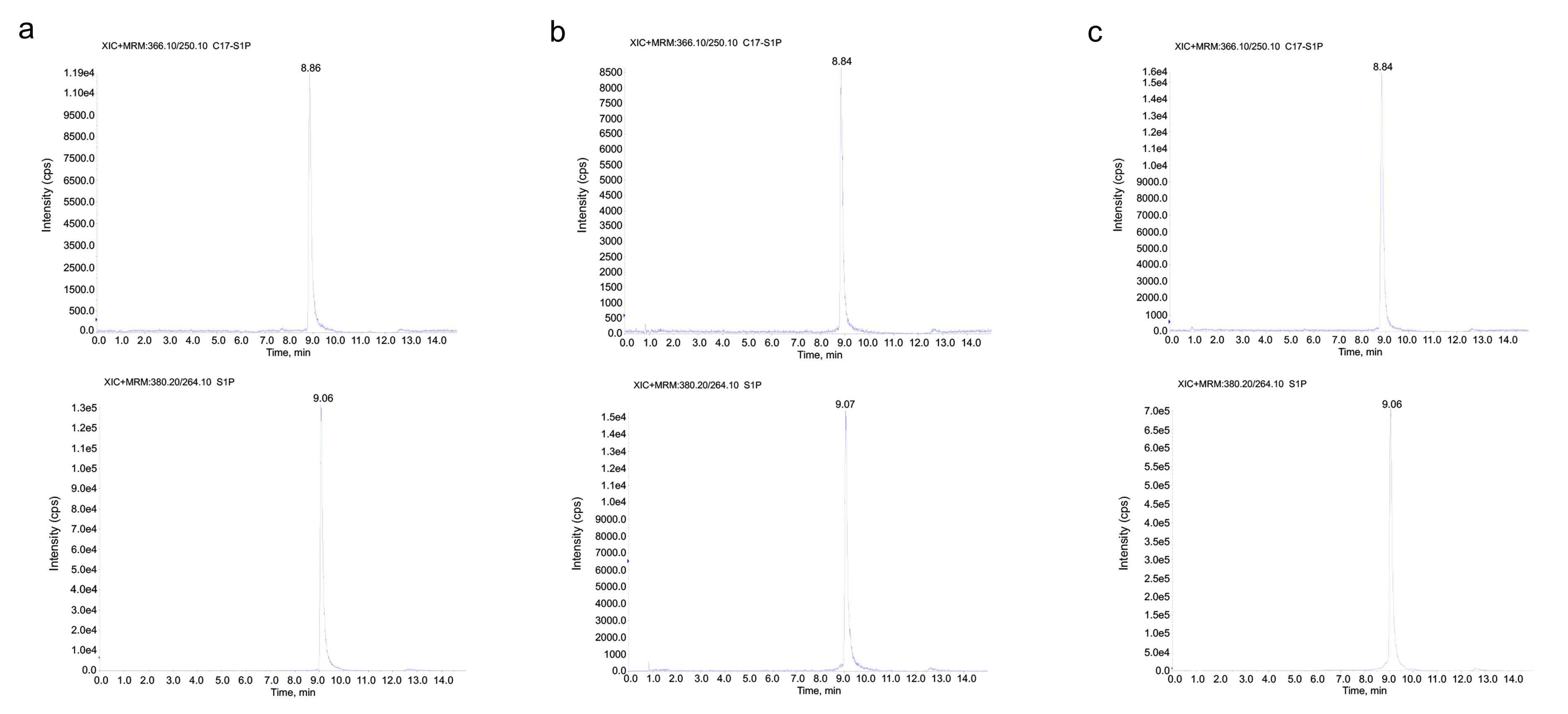

Supplement: Supplementary file 12 — Figure S10 [file 41419_2021_3848_MOESM12_ESM.tif]

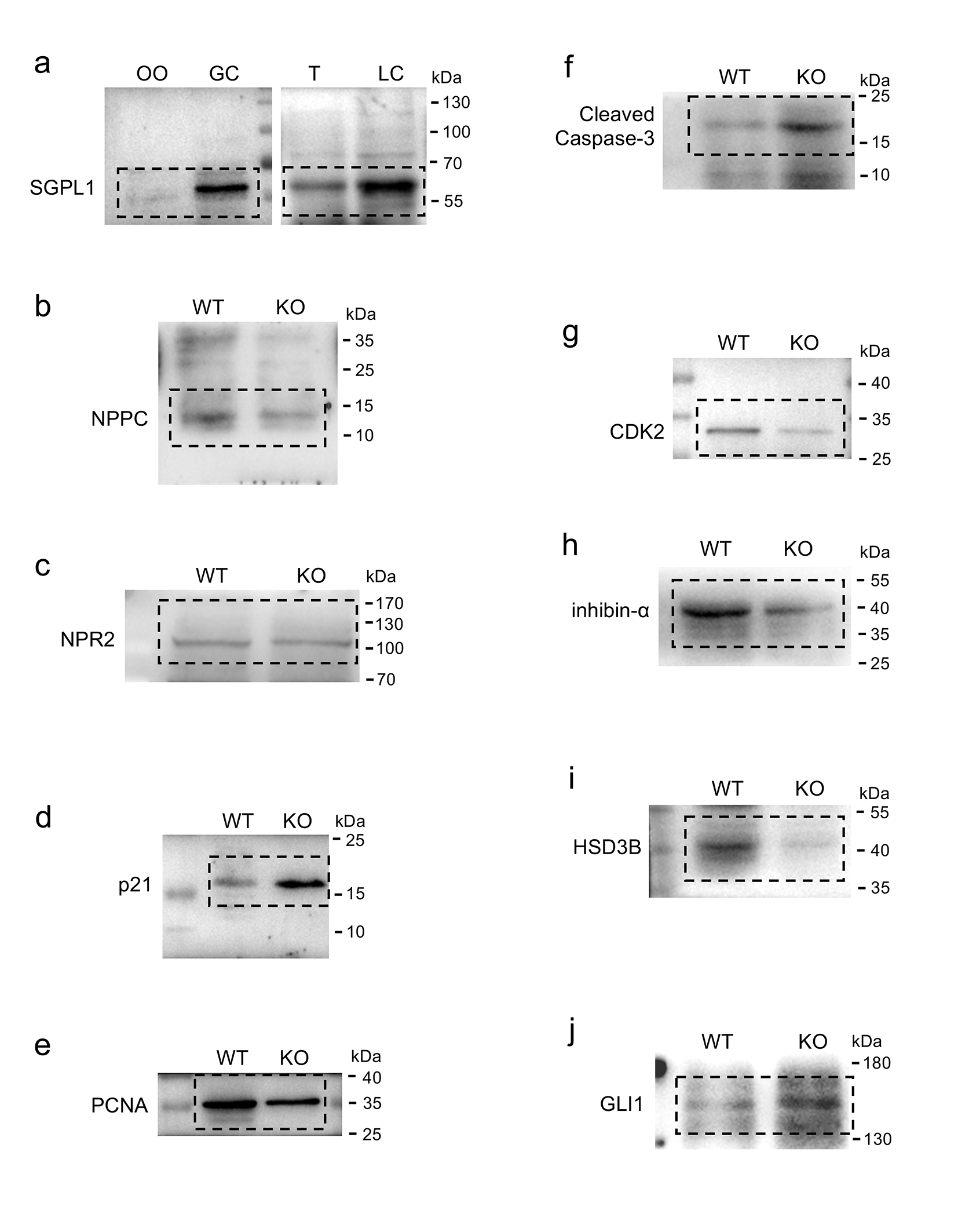

Supplement: Supplementary file 13 — Figure S11 [file 41419_2021_3848_MOESM13_ESM.tif]
